# Genome reconstruction in *Cynara cardunculu*s taxa gains access to chromosome-scale DNA variation

**DOI:** 10.1038/s41598-017-05085-7

**Published:** 2017-07-17

**Authors:** Alberto Acquadro, Lorenzo Barchi, Ezio Portis, Giulio Mangino, Danila Valentino, Giovanni Mauromicale, Sergio Lanteri

**Affiliations:** 1 DISAFA, Plant Genetics and Breeding, University of Torino, Torino, Italy; 2 Di3A, Dipartimento di Agricoltura, Alimentazione e Ambiente, University of Catania, Catania, Italy

## Abstract

The genome sequence of globe artichoke (*Cynara cardunculus* L. var. *scolymus*, 2n = 2x = 34) is now available for use. A survey of *C*. *cardunculus* genetic resources is essential for understanding the evolution of the species, carrying out genetic studies and for application of breeding strategies. We report on the resequencing analyses (~35×) of four globe artichoke genotypes, representative of the core varietal types, as well as a genotype of the related taxa cultivated cardoon. The genomes were reconstructed at a chromosomal scale and structurally/functionally annotated. Gene prediction indicated a similar number of genes, while distinctive variations in miRNAs and resistance gene analogues (RGAs) were detected. Overall, 23,5 M SNP/indel were discovered (range 6,34 M –14,50 M). The impact of some missense SNPs on the biological functions of genes involved in the biosynthesis of phenylpropanoid and sesquiterpene lactone secondary metabolites was predicted. The identified variants contribute to infer on globe artichoke domestication of the different varietal types, and represent key tools for dissecting the path from sequence variation to phenotype. The new genomic sequences are fully searchable through independent Jbrowse interfaces (www.artichokegenome.unito.it), which allow the analysis of collinearity and the discovery of genomic variants, thus representing a one-stop resource for *C*. *cardunculus* genomics.

## Introduction

The genus *Cynara*, a member of Asteraceae family (a.k.a. Compositae), contains eight species and four subspecies, all native to the Mediterranean basin^1^. The members of the species *Cynara cardunculus* L. (2n = 2x = 34) are globe artichoke (var. *scolymus* (L.) Fiori), cultivated cardoon (var. *altilis* DC.) and the wild cardoon (var. *sylvestris* (Lamk) Fiori). The three *C*. *cardunculus* botanical varietas are fully cross-compatible with one another, and their F_1_ hybrids are fully fertile. This, together with phenotype data^2^ and inferences based on genetic markers^3, 4^ suggest that both the globe artichoke and cultivated cardoon were domesticated from wild cardoons and it is likely that their domestication occurred in the island of Sicily^5^. Globe artichoke was anthropogenically selected for the production of immature inflorescences (heads or capitula), and cultivated cardoon for its fleshy stalks^4^.

The three *C*. *cardunculus* taxa are also exploited for the production of a number of nutraceutically and pharmaceutically active compounds^6–11^ such as phenylpropanoids (mono- and di- caffeoylquinic acids) and sesquiterpene lactones, the latter being responsible for their characteristic bitter taste. Cultivated cardoon in particular represents a source of both lignocellulosic biomass^12–14^ and seed oil for edible and biofuel uses^15–18^.

After Italy, the next biggest globe artichoke producers are Egypt and Spain (FAO^19^ data, 2013), but its cultivation has spread to the Near East (Turkey and Iran), North Africa (Algeria, and Tunisia), South America (Argentina, Chile and Peru), United States (mainly in California) and China. Italy also harbors the richest primary cultivated gene-pool, which as a rule is classified on the basis of capitulum traits^20–22^ into: i) *‘Spinosi’*: with long sharp spines on bracts and leaves; ii) *‘Violetti’*: with medium-sized, green-violet-colored capitula and fleshy thorns; iii) *‘Romaneschi’*: with spherical or sub-spherical green capitula and; iv) *‘Catanesi’*: with relatively small, elongated capitula with more or less marked violet streaks.

Recently, we published the sequence of the globe artichoke genome^23^ (www.artichokegenome.unito.it). Its assembly, comprising 13,588 scaffolds covering 725 of the 1,084 Mb genome, was generated using ∼133-fold Illumina sequencing data and encodes 26,889 predicted genes. A new highly saturated linkage map was also constructed, which represents a big step forward from the genetic maps we previously developed^24–27^.

In the present work we report on the re-sequencing analysis of four globe artichoke genotypes (SP, C3, VS and VT) representative of the core varietal types in cultivation and also a genotype of cultivated cardoon, A41. By combining iterative mapping and reference guided-assembly, the five genomes were reconstructed and annotated; miRNA loci as well the number, position and phylogenetic relationships of putative resistance gene analogues RGAs were identified. Finally, SNPs/indels among the five genotypes were highlighted versus the reference globe artichoke genome, and a functional SNP analysys was carried out on the metabolic pathways of phenylpropanoids and sesquiterpene lactones.

## Results

### Genome assembly and reconstruction

Genome sequencing of six *C*. *cardunculus* genotypes (Table 1) yielded 1.4 billion raw pair-end reads with an average length of 100 bp (Table 2). The latter were reduced to 1.27 billion (94%) after filtering/trimming for high quality reads, corresponding to a total of 395.7 Gbp available for the assembly procedures. The sequence depth of coverage ranged from 24.6× (A41) to 45.3× (SP), being 35.3× on average (Table 2). A genome reconstruction method was adopted based on a combination of iterative read mapping against the globe artichoke reference together with *de novo* assembly. *De novo* assembly was performed using k-mers that allowed achieving the best assembly for each genotype. The values k = 68 and k = 66 were selected for A41 and C3 respectively, with k = 65 for SP, VS and VT. The contig-based assemblies was then used as a basis for the genome reconstructions of the five genotypes (Table 2).

**Table 1:** Details of the *Cynara cardunculus* genotypes studied.

Accession name	Group	Code	Propagation
‘2 C’ (reference)	Artichoke breeding line	2 C	seed
‘Altilis 41’	Cultivated cardoon	A41	seed
‘Violetto di Sicilia’	Catanesi	VS	shoot
‘Violetto di Toscana’ (‘Tema’)	Violetti	VT	seed
‘Romanesco C3’	Romaneschi	C3	shoot
‘Spinoso di Palermo’	Spinosi	SP	shoot

**Table 2:** Genomics statistics.

Sequencing data	2 C	A41	VS	VT	C3	SP
SRA codes	SRR1914377; SRR1914378	SRR1826176; SRR1826114; SRR1914331	SRP055806	SRP055806	SRR1826175; SRR1825940; SRR1914330	SRP055806
Number of raw reads	—	90,410,254 (×2)	148,872,150 (×2)	129,452,237 (×2)	126,585,508 (×2)	174,120,908 (×2)
Number of reads	—	88,593,112 (×2)	138,616,098 (×2)	121,283,190 (×2)	123,535,166 (×2)	163,030,615 (×2)
Total amount sequence (Gb)	—	110	82,1	64	46	93,6
Estimated fold coverage	—	24.6×	38.5×	33.7×	34.3×	45.3×
**ABySS assembly**						
Number of contigs	—	5,741,441	6,988,492	6,242,434	8,456,162	7,566,149
Total length (contigs, Mb)	—	1,106.4	1,001.4	922.1	1,409.1	1,116.3
**IMR-DENOM reconstruction**						
Number of sequences	79,681	95,970	74,740	74,498	77,535	74,317
Sequences/Mb	121.6	147.2	115.7	115.5	118.7	115.3
Total length (contigs, Mb)	654.6	651.6 (99,5%*)	645.9 (98,6%*)	644.7 (98,4%*)	652.8 (99,7%*)	644.3 (98,4%*)
Total length (scaffold, Mb)	724.7	721.9 (99,6%*)	714.6 (98,6%*)	713.1 (98,4%*)	722.9 (99,7%*)	712.3 (98,3%*)
L50 (Kb)	17.5	13.5	8.9	8.9	9.5	8.9
N50	10,596	13,964	20,425	20,491	19,621	20,504
L90 (Kb)	3,4	1,3	1,3	1,3	1,4	1,4
N90	41,711	46,781	46,036	45,970	45,776	45,799
G + C %	32.00%	35.18%	35.04%	35.08%	35.28%	35.01%
N° of sequences > 10 Kb	20,561	20,897	19,975	19,915	20,454	19,922
**Number of genes**	28,310	27,785	27,121	27,160	28,029	27,326
**Number of proteins with IPR**	22,571 (79.7%)	22,199 (79.9%)	21,898 (80.7%)	21,888 (80.6%)	22,406 (80%)	21,997 (80.5%)

Sequencing (Illumina), assembly (ABySS-based^91^), genome reconstruction (IMR/DENOM^41^) and gene prediction statistics of the A41, VS, VT, C3 and SP genotypes. The reference genome (2 C) data of the libraries are available in Scaglione *et al*.^23^. *Percentage of reconstructed genome compared with the 2 C genome.

### Genome annotation and OrthoMCL analysis

**Figure 1:**
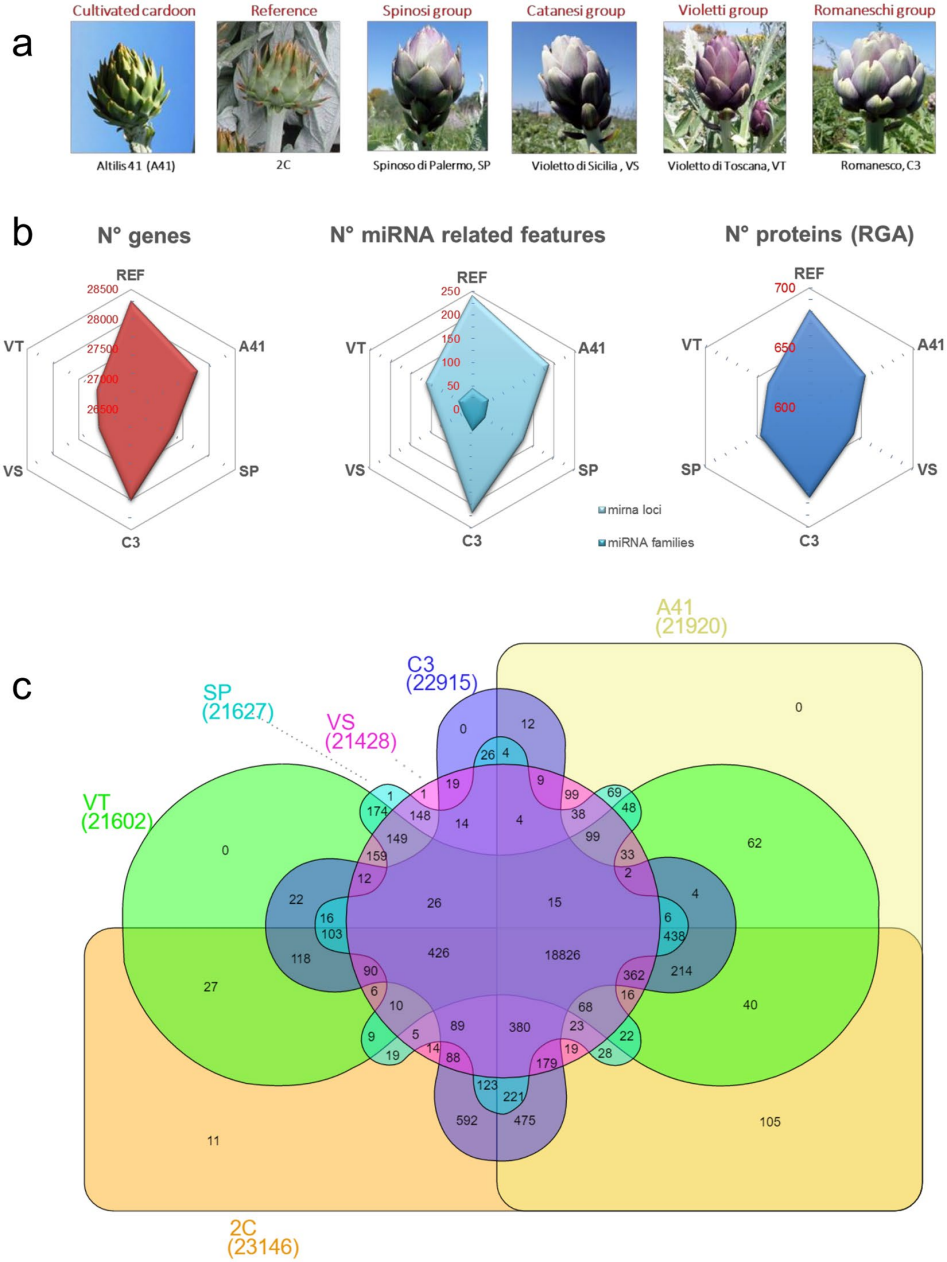
Feature content analysis in the sequenced genomes of *C*. *cardunculus*. (**a**) Heads of the six analyzed genotypes (REF, VT, SP, VS, A41, C3. (**b**) Relative number of predicted genes, miRNAs loci and total RGAs loci. (**c**) Distribution of orthologous gene families in 2 C (reference), VT, SP, VS, A41 and C3 genotypes, calculated using OrthoMCL^29^.

Gene prediction was conducted for the five re-sequenced genotypes and a fresh gene prediction was also performed for the reference genome (2 C), which increased the gene number from 26,889^23^ to 28,310. The six genomic sequences, together with their structural and functional annotations are available at www.artichokegenome.unito.it, and they are fully searchable through six independent Jbrowse^28^ interfaces. Comparative analysis revealed a similar number of genes for all the genotypes under study (Fig. 1, Table 2), with 2 C and VS having the highest (28,310) and lowest (27,121) number of genes (AED ≤ 0.5) respectively. Orthology between genes was also assessed via a reciprocal best-hit analysis (File S1).

OrthoMCL^29^ clustered 161,855 sequences from the six genotypes into 24,417 gene families (excluding singletons) (Fig. 1 and Table S1). A total of 18,826 gene families (containing 138,098 genes) were in common between the six genotypes, while 426 (with 2145 genes) were shared between the five globe artichoke genotypes. All in all, just 11 gene families (24 genes) were absent from the reference genome while 1 (2 genes) was unique to VS and SP. The non-spiny genotypes (VS, VT and C3) shared 12 gene families (including 36 genes), with the ‘pigmented’ types VS and VT sharing 159 gene families (including 318 genes). It was found that 149 gene families (449 genes) were specific to the oblong capitula types (SP, VS and VT). Lastly, the number of sequences not falling into any cluster (singletons) ranged from 149 (in the reference 2C) to 999 (in the cultivated cardoon A41).

A search for enrichment analyses (SEACOMPARE^30^) for genes shared between the five artichoke genotypes (Table S2), revealed significant enrichment for some GO^31^ terms. The top ranked enrichments observed for processes were GO:0006412 (translation) and GO:0010467 (gene expression), the latter missing in C3 genotype. With respect to functions (F), enrichments were observed in GO:0005198 (structural molecule activity) and GO:0003735 (structural constituent of ribosome). For components (C), enrichments were present for GO:0005840 (ribosome) and GO:0030529 (ribonucleoprotein) complexes. InterProScan^32^ analyses highlighted about 80% of the predicted proteins with at least one IPR domain (Table 2). The top 20 SUPERFAMILY^33^ domains are listed in Table S3 and the most abundant domain was SSF52540 (P-loop containing nucleoside triphosphate hydrolase) which is involved in several UniPathways, including chlorophyll or coenzyme A biosynthesis. The second most abundant SUPERFAMILY ID was SSF56112 (protein Kinase-like domain), which includes proteins acting on signaling and regulatory processes in the eukaryotic cell, followed by SSF52058 (Leucine-rich repeat domain, L domain-like) and SSF48371 (Armadillo-type fold), which are involved, *inter alia*, in defense response and translation factor activity respectively.

### Detection of Presence/Absence variants (PAVs)

Based on gene coordinates, the five datasets were inspected for PAVs and 346 putative PAV genes (Figure S1) was uncovered. The frequency of PAVs varied among the genomes ranging from 241 in SP and VS to 225 and 261 in C3 and VT respectively. Among the 251 shared PAVs, 173 (50.0%) were absent in all the genotypes, while 30 (8.7%) were absent in 2 genotypes and 14 (4.1%) in three genotypes. A total of 87 (25.1%) of the detected PAV genes were absent only in one genotype (17 in SP, 14 in VS, 4 in C3, 27 in VT and 25 in A41). The PAV genes exclusively present in just one accession were 17 in A41, 1 in SP, 4 in C3, 1 in VS and 2 in VT (Figure S1, Table S4). Functional information for candidate specific absent and present PAV genes was evaluated (Supplementary data) and a GO enrichment analysis conducted for PAV genes (17) present in the genotype A41 (Table S5).

### Prediction and annotation of miRNA

From a search against miRBase^34^, 21 high confidence database, species-specific miRNAs were predicted and used for further analyses (File S2). The total number of predicted non-redundant miRNAs varied from 51 (within 74 VS genome regions) and 143 (in 241 genome regions of the reference 2 C), belonging to 32 (for SP) or up to 45 (2 C) miRNA families (Fig. 1 and Table S6). The Tapir hybrid^35^ search for target genes of the identified miRNA in the six genotypes, revealed between 307 (VS) and 1167 (2 C) putative miRNA: mRNA duplexes. Almost 90% of genes encoding predicted target transcripts have functional InterPro annotations. The total number of miRNA families involved in miRNA: mRNA interactions varied according to the genotype, ranging from 20 in VS to 45 in 2 C and C3 (Fig. 1 and Table S7). Although the main families involved in miRNA: mRNA duplex formation were generally genotype-specific (Table S8), miRNA172 was the top ranked family for all the genotypes (varying between 83 for VS and 283 for C3), with the exception of SP where no miRNA 172 were identified. Putative miRNA-target gene enrichment analysis for each genotype revealed significant enrichment for some GO terms (Supplementary data, and File S3). The REVIGO^36^ summarization of enriched terms for biological process, cellular component and molecular function, obtained by removing redundant GO terms, are reported in Figure S2, S3 and S4. Finally, comparisons of GO term enrichment (AGRIGO SEACOMPARE^30^) applied to the five genotypes showed that just one GO term (GO:0005634: nucleus) was shared among all the genotypes (Table S9). As previously reported^23^, miRNAs are predicted to target known transcription factors related to plant development, morphology and flowering time. Examples include miR160 and ARF (Auxin Response Factor: absent in VS), miR156 and SQUAMOSA promoter binding-like proteins, miR164 and NAC-like proteins (absent in VT and SP), miR172 and AP2-like proteins (absent in SP) and miR171 and GRAS-like proteins (absent in VS).

### Resistance genes

A total of 682 proteins in 2 C, 654 in A41, 643 in VS, 675 in C3, 647 in SP and 640 in VT (Fig. 1), showed homology (at p < 1e-60) with 1,605 out of the 2,680 *Arabidopsis* unique RGA proteins. The Hmmer^37, 38^ search of HMMs for key resistance genes motifs revealed that in all genotypes, about 89% of the RGAs identified by Blastp against *Arabidopsis* unique RGA proteins contained at least one HMM domain (File S4). The majority of RGAs (Table 3) belonged to the RLK family (170 in VT up to 180 in C3), followed by proteins containing P-kinase and TM domains (other-KTM), ranging from 146 in A41 to 157 in 2 C, and RLP, with 65 in both VS and SP up to 86 in 2 C). In addition, a few RGAs containing at least one NB-ARC domain were identified (Table 3).

**Table 3:** Classification of RGAs identified in the six genotypes.

**Identified Domains**	**Acronym**	**2 C**	**A41**	**VS**	**VT**	**C3**	**SP**
LRR:NB-ARC	NL	9	7	7	6	8	7
LRR:NB-ARC:CC	CNL	4	4	4	6	4	5
LRR:NB-ARC:TIR	TNL	—	—	1	—	—	—
LRR:NB-ARC:TM	other-TMNL	1	—	1	—	1	—
LRR:NB-ARC:TM:CC	other-LNTMC	—	—	—	—	—	1
NB-ARC	N	9	8	9	8	9	11
NB-ARC:CC	CN	7	7	8	8	8	10
NB-ARC:TIR	TN	1	1	1	1	1	1
NB-ARC:TM	other-NT	4	3	3	2	4	2
NB-ARC:TM:CC	other-NTMC	3	2	1	2	3	1
LRR:Pkinase:TM	RLK	173	175	175	170	180	173
LRR:TM	RLP	86	72	65	71	77	65
Pkinase	other-K	59	58	57	54	58	60
Pkinase:CC	other-KC	9	9	8	9	9	8
Pkinase:TM	other-KTM	157	146	149	156	155	148
Pkinase:TM:CC	other-KTMC	4	4	3	3	4	5
LRR:Pkinase	KL	1	3	2		1	1
LRR:Pkinase:TM:CC	other-LRTMC	5	5	4	6	6	6
LRR:TM:CC	other-CLTM	1	—	—	—	—	—
TIR	T	2	2	—	2	2	2
LRR	L	22	27	24	17	19	23
ABC_tran	other-A	1	1	1	2	1	2
ABC_tran:Pkinase:TM	other-AKTM	—	1	—	—	—	—
ABC_tran:TM	other-ATM	49	46	46	47	50	46
ABC_tran:TM:CC	other-ACTM	4	4	5	4	3	4
**Total**	—	611	585	574	574	603	581
% of Blastp genes identified with HMM domains	—	0,9	0,9	0,9	0,9	0,9	0,9
Blastp genes identified	—	685	654	643	640	675	647

RGA proteins classified based on domain identification together with their frequency.

**Figure 2:**
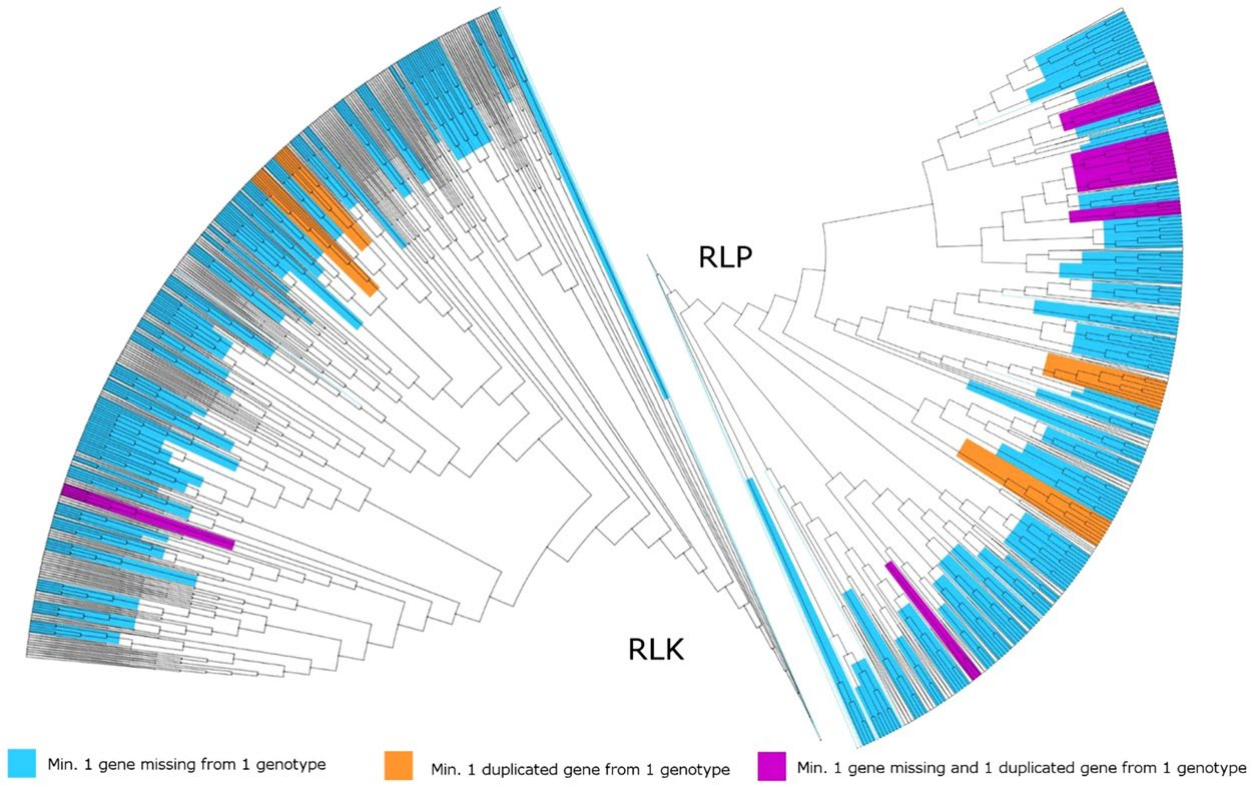
RLP and RLK resistance genes dendrogram of the six *C*. *cardunculus* genotypes. Taxa missing one or more RGA gene from at least one genotype are highlighted in light blue; clusters with one or more duplicated genes in one genotype are highlighted in orange, while clusters with at least one gene missing from one genotype and also containing at least one duplicated gene from one genotype are highlighted in purple.

The number of RLK, RLP, NB and other-KTM genes detected in each chromosome of the six genotypes in study is shown in Figure S5. In all the genotypes most RGAs were found on chromosome 1, followed by chromosomes 2, 13, 10 and 5. Chromosomes 6, 7, 14 and 16 (with the exclusion of C3) were devoid of RLP genes, while chromosomes 3, 6, 8 (with the exclusion of C3), 11, 12, and 14 were without NB genes. Distinct clusters of RGAs were identified for three of the main classes: RLK, RLP, NB and other-KTM (Table S10). In particular, chromosome 10 was found to harbor two RGA clusters: one of genes with an NB domain in a region of two Mb (16–18 Mb) and one of other-KTM genes at 10–11 Mb. No clusters of RLK genes were identified. RGAs were found to be quite uniformly distributed along the 17 chromosomes, except for the clustering of RLP genes on ch.1 (0–3 Mb) and ch.11 (1–3 Mb), of NB genes on ch.13 (40–41 Mb), and of other-KTM genes on ch.10 (10–11 MB). Alignments of the amino acid sequences and subsequent RAxML^39^ analyses allowed the generation of phylogenetic trees for each of the RGA classes in the study. As expected, each resistance gene and its orthologs clustered together, although in some taxa one or more orthologs were absent (File S5). In particular for the most numerous RGA families, RLK and RLP, taxa missing at least one RGA from at least one genotype were 46 (24%) and 45 (50%) respectively (Fig. 2 light blue). In addition, 2 clusters for both RLKs and RLPs contained at least one duplicated gene from one genotype (Fig. 2 orange), while clusters with at least one missing gene from a genotype and a duplicated gene from another genotype amounted to 1 for RLK and 4 for RLP (Fig. 2, highlighted in purple).

### SNP/indel discovery and heterozygosity estimation

**Figure 3:**
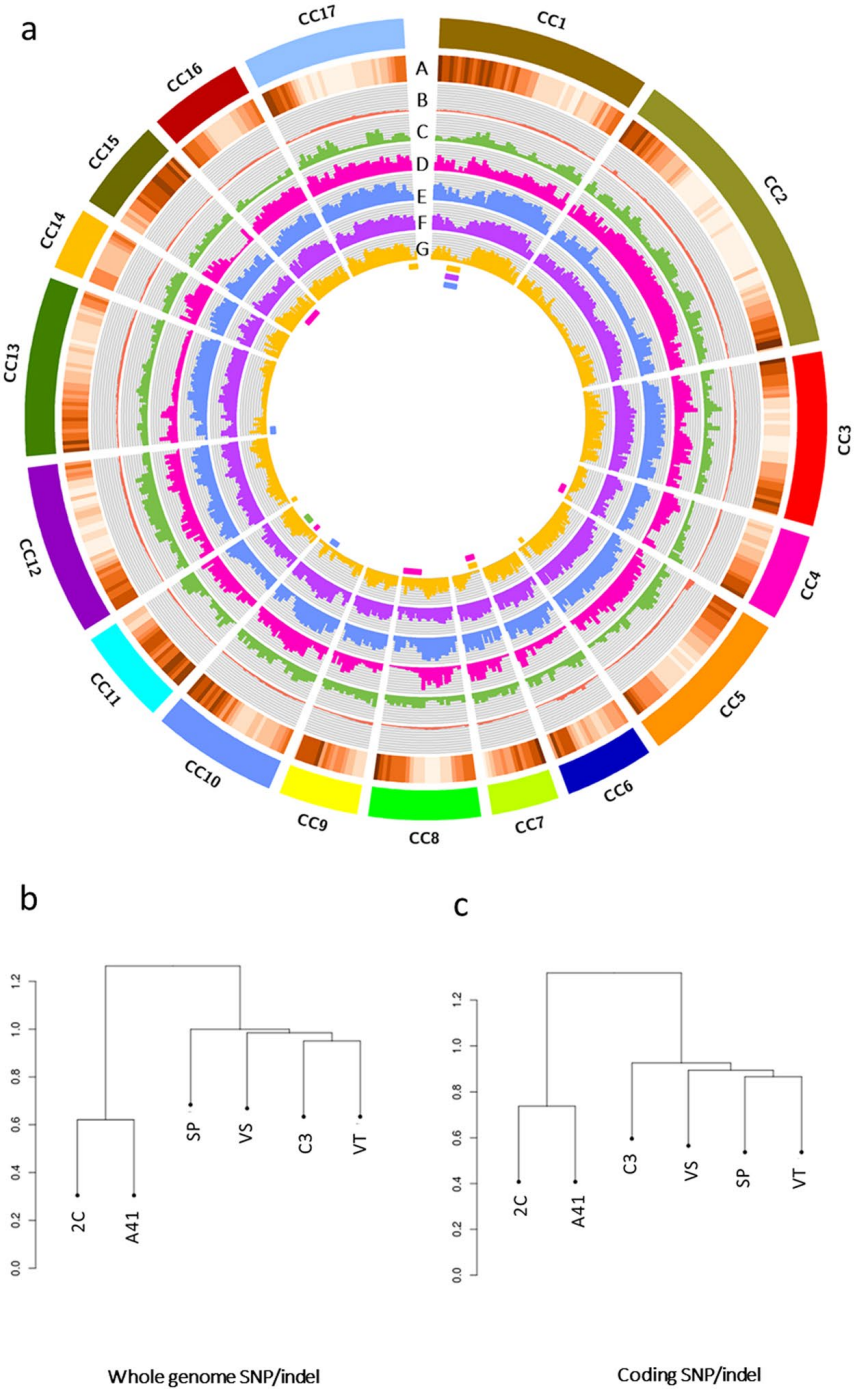
Representation of the existing genetic variability among the *C*. *cardunculus* genotypes analyzed. (**a**) Circos diagram depicting gene and heterozygous SNP densities; from the outer circle to the inner circle: A) Heat map of gene density in the reference genome; 1 M histograms representing the density of heterozygous SNPs for the Reference (track B) A41 (track C), SP (track D), VS (track E), C3 (track F), VT (track G). (**b**) UPGMA-based dendrograms of the six genotypes taking into account 23 M SNPs and (**c**) 500 k coding SNPs.

To identify large-scale polymorphisms of sequenced accessions, reads were aligned against the globe artichoke reference genome (2 C). The mapping rate across different accessions varied from 95.8% to 97.6%, for an average of 96.5%. The whole SNP/indel set contained 23,450,539 entries (File S6), with 815,853 in 2 C rising to 14,495,680 in VS (Table 4). In the sequenced globe artichoke genotypes, similar SNP/Indel numbers were detected in SP and VS (ca 14.4 M) with slightly fewer in C3 and VT (ca 12.8 M), while in the cultivated cardoon A41 SNP/Indels halved to about 6 M. Among the globe artichoke genotypes the highest SNP frequency was found in VS (1/54 bp) with the highest indel frequency in SP (1/122 bp). Table S11 reports heterozygosity levels estimated across the six genotypes. All the genotypes, except the reference (2 C) which has been specifically bred by repeated cycles of selfing to attain a very low level of residual heterozygosity (Fig. 3a, track B), showed SNP-dense regions dispersed genome-wide (Figure S6). However, many regions with a low frequency of heterozygous SNPs (Fig. 3) and carrying SNPs fixed in the homozygous state (Figure S6) were observed. Some of them were genotype-specific (Fig. 3a) and occurred in gene-dense regions: chromosomes 4, 8, 11, 15 in SP; ch.10, 13 in VS; ch.5, 12, 17 in VT; ch.11 in A41. Some others are in common between genotypes (ch.1 in VS, C3 and VT). Interestingly, in chromosome 7 a wide region carrying SNPs mainly in the homozygous state was observed in both the SP and VT genotypes. Genetic relatedness among the six genotypes was assessed based on the whole SNP set (23 M) and also the SNPs detected in coding sequences (Fig. 3b). In both cases the reference genotype (2 C) and the cultivated cardoon (A41) clustered at a high level of genetic differentiation from the four globe artichoke genomes. Small differences were detected in the relationships among the latter, as the whole genome SNP analysis revealed a higher similarity between VT and C3, and a significant genetic differentiation between spiny (SP) and non-spiny (VS, VT and C3) globe artichoke types, while the coding SNPs highlighted a higher-similarity VT/SP cluster.

**Table 4:** Statistics (SNP, Indel and SNP/indel) of the analyzed genotypes.

Genotypes	2 C	A41	VS	VT	C3	SP
SNP	781,530	5,900,934	13,440,135	11,860,358	11,937,400	13,241,315
SNP rate	0.11%	0.81%	1.85%	1.64%	1.65%	1.83%
SNP/1000 bp	1.07	8.13	18.53	16.35	16.46	18.26
1 SNP every (bp)	927.6	122.8	53.9	61.1	60.7	54.7
Indel	34,232	443,603	1,055,545	987,949	910,23	1,150,341
Indel rate	0.0047%	0.06%	0.15%	0.14%	0.13%	0.16%
Indel/1000 bp	0.05	0.61	1.46	1.36	1.26	1.59
1 Indel every (bp)	21,179	1,634	686.8	733.8	796.5	630.2
SNP/indel	815,853	6,344,545	14,495,680	12,848,307	12,847,630	14,391,656
SNP/indel rate	0.11%	0.87%	1.99%	1.77%	1.77%	1.98%
SNP/indel/1000 bp	1.12	8.75	19.99	17.72	17.72	19.85
1 SNP/indel every (bp)	892.85	114.27	50.01	56.42	56.43	50.37

### Variants annotation

**Figure 4:**
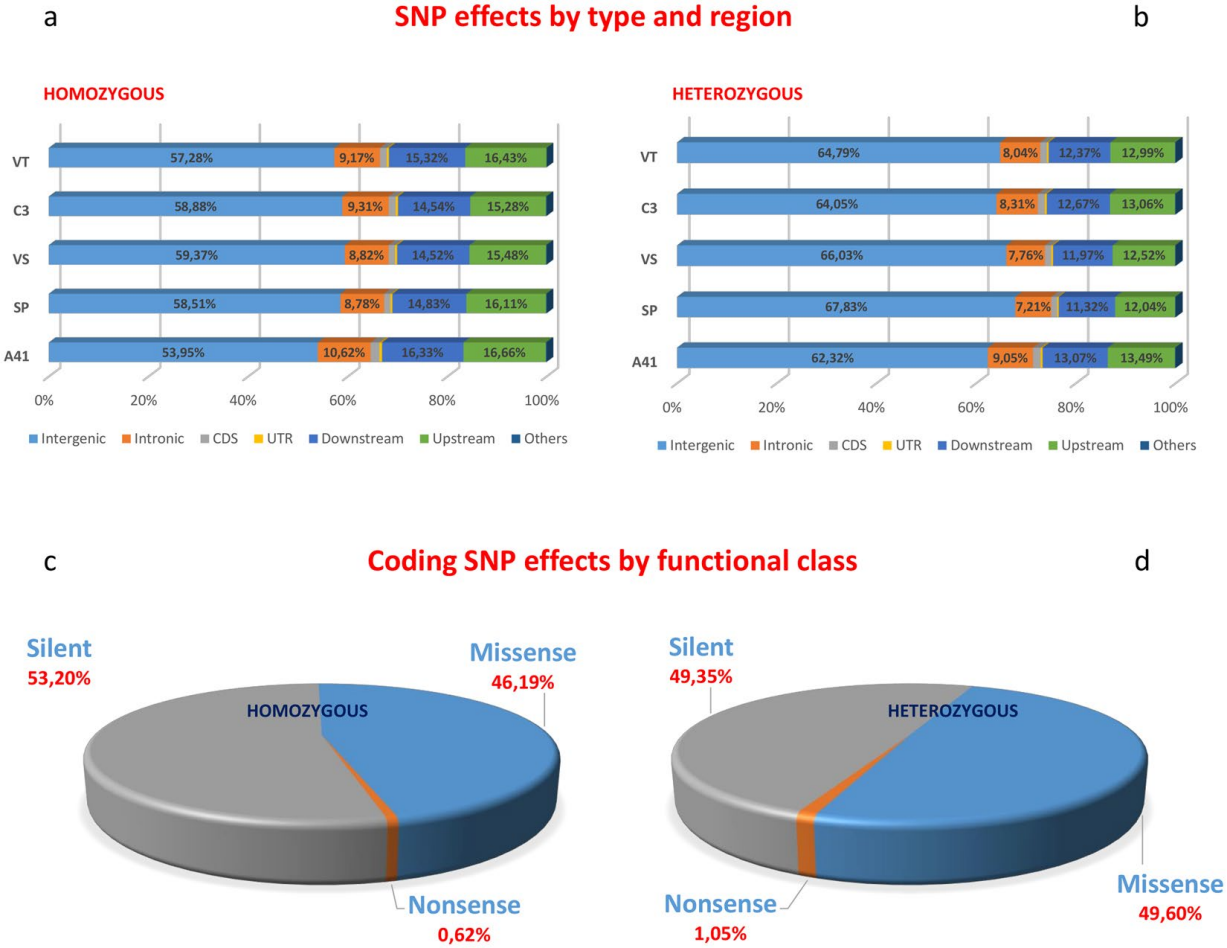
SNP categorization of coding and non-coding variants. SNP effect by type and region for homozygous (**a**) and heterozygous (**b**) regions. SNP effect by functional class for homozygous (**c**) and heterozygous (**d**) regions.

The analysis of functional variants between the reference and the resequenced genotypes was performed using the SnpEff^40^ suite on both heterozygous and homozygous SNP/indel sets. The observed fraction of coding SNPs (500 k, exon-based) was as little as 2% of the 23 M detected SNPs. Two thirds of the total homozygous variants were located outside the gene space (intergenic region: 57.6%; intronic region: 9.3%) with only a small fraction contained within coding sequences (1.41%, Fig. 4). In SP a total of 6.2 M mutations were detected, 5,290,997 were observed as intergenic (Table S12), while 113,099 were in the annotated gene space. VS and VT showed a similar number of intergenic mutations, while variants annotated in CDS were 96,437 and 108,712 respectively. In C3, 3,719,864 intergenic SNPs were found, with 89,177 SNPs located in genic space. Finally, A41 (intergenic mutations: 1,470,424) contained 47,438 SNPs in CDS. About 98% of the variants were classified as modifiers. The fraction of moderate variants ranged from 0.58% to 0.8% according to accessions, and those having low effect from 0.75% to 1.05%. The high effect variants were the smallest class, with 1,464 to 3,761 mutations depending on the specific genotype (Table S12). Among the homozygous variations detected in coding sequences, 53.20% led to synonymous and 46.19% to non-synonymous amino acid changes, while 0.62% gave rise to non-sense mutations (Table S13). In respect to the heterozygous SNP/indels, the highest number of variants were mutations located in the intergenic and intronic regions (65.0% and 8.1%), while only 1.35% occurred in genic space (CDS, Fig. 4, Table S12). The high effect variants represented only 0.048% of total mutations. The fraction of mutations with low effect varied between 0.65% and 0.86%, while moderate variants accounted for between 0.6% and 0.77%. About 98% of the total mutations in all the resequenced genotypes were classified as modifier variants. With regard to the variations detected in coding sequences, 49.60% were annotated as non-synonymous mutations. Synonymous mutations amounted to 49.35% of total variants and only 1.05% were classified as non-sense mutation (Table S13).

### Analysis of variants impact on CQAs and SLs gene classes

**Figure 5:**
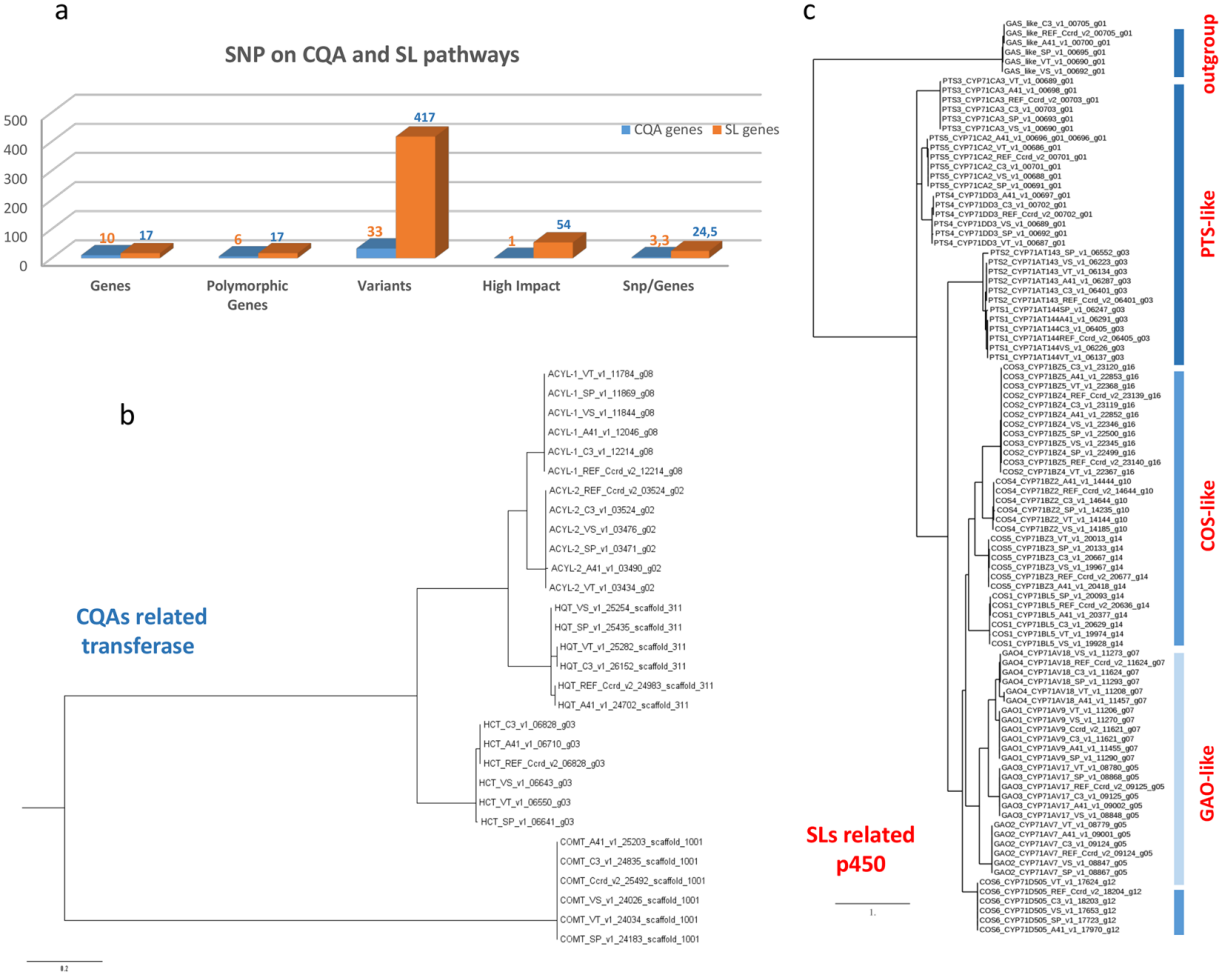
Coding SNPs data in CQAs and SLs pathways. (**a**) Number of genes, polymorphic genes, variants and high deleterious impact SNPs in CQA- (light blue) and SL- (orange) related genes. Phylogenetic trees of proteins belonging to CQA-related transferase genes from the six genotypes analyzed (**b**) and to SL-related p450 genes from the same accessions (**c**).

The possible impact of variants localized in genes responsible for caffeoylquinic acids (CQAs) and sesquiterpene lactones (SLs) biosynthesis were analyzed. Almost all investigated genes showed variants, with SLs showing a higher number of variants (24.5 SNP/gene) compared to CQAs (5.5 SNP/gene, Fig. 5). The genes with non-synonymous variants potentially giving rise to high-effect impacts are reported in Table S14. Of the 33 non-synonymous variants belonging to the CQAs related genes, 10 (30.3%) showed a predicted deleterious effect in coding sequences when translated as amino acid substitution. These were located in all the polymorphic genes (Fig. 5). In the SL group, which contained 417 non-synonymous variants, about 83 (19.9%) were deleterious. All the coding SNPs were in the heterozygous state and affected 14 of the 17 genes (Fig. 5). In the CQAs pathway, one significant deleterious mutation, which creates a premature stop-codon, was heterozygous in the C4H gene (in SP, Table S14). No relevant homozygous deleterious variations were identified in the SL gene class. However, many deleterious variants in the heterozygous state were found in the germacrene A synthase (GAS) gene, which mutated exclusively in the four globe artichoke genotypes, and not in the cultivated cardoon (A41, Table S14). The impact of coding SNPs on protein genetic diversity among the analyzed genotypes is shown using family based phylogenetic trees (Fig. 5).

## Discussion

Among the species belonging to the Asteraceae family, *Cynara cardunculus* has remained for a long time relatively unexplored compared to other species such as sunflower and lettuce, for which genomes extensive investigations have been performed. Following our recent release of the first globe artichoke genome sequence^23^, we performed the first WGR (at ~35× coverage) of five *C*. *cardunculus* genotypes, four representatives of the core globe artichoke varietal types at present in cultivation and one of the related and inter-fertile *taxa* cultivated cardoon. By the use of a genome reconstruction strategy based on iterative mapping and reference-guided assembly^41^, the five genomes were assembled and reconstructed at the chromosome scale. Our approach proved to be less demanding in terms of sequencing depth and multiple libraries construction compared to a *de novo* assembly. The sizes of the reconstructed genomes were comparable to that of the reference genome, with an average percentage of reconstruction close to 98.9%, but smaller compared to the estimated genome size of *C*. *cardunculus* (1,084 Mbp). This is likely due to the absence in the reference genome assembly of some repetitive sections, though it included about 95% of the gene space^23^. Indeed, by performing a new gene prediction of the globe artichoke reference sequence following the application of a more stringent AED threshold, we found 28,310 predicted genes, which corresponds to a 5% increase over the 26,889 previously predicted.

As reported for other species^42^, globe artichoke and cultivated cardoon suffer major losses from numerous diseases, as a result of many years of cultivation and selection mainly focused on desirable commercial traits at the expense of disease resistance. Plants have developed effective mechanisms to recognize and respond to infections caused by pathogens, among these the RGAs^43–45^ play a key role. In the five reconstructed genomes in study, as well as in the reference genome, we computationally mined and characterized RGAs on the basis of their significant structural features and conserved domains.

The RGAs identified here represent on average about 2% of the total number of predicted genes for all the genotypes. The majority of them were located preferentially on six chromosomes: 1, 2, 3, 5, 10 and 13, suggesting their specialization in resistance pathways. Their location assists development of a high-density genome-wide RGA genetic map for the species, which is pivotal for designing diagnostic markers and identifying quantitative trait loci (QTL) or markers associated with plant disease resistance. *Cynara cardunculus* RGAs mainly fall into RLK, RLP and other-KTM (i.e. genes with a P-kinase and a TM domain) classes (Table 3), while NBS (such as TNL, CNL, and TN) were poorly represented. Previous studies report that TN (TIR-NBS) are the largest group of resistance genes in both *Arabidopsis thaliana* (64%)^46^ and *Brassica rapa* (64%)^47^, but they are rare in *Oryza sativa* (1%)^48^ and *Sorghum bicolor* (1%)^49^ and absent in *Brachypodium distachyon* and *Zea mays* ^50^, suggesting their specificity for dicotyledons^51^. Kim *et al*.^52^, in a survey of RGA genes based on UNIGENE analyses, highlighted that some Asterids, such as *Solanaceae*, contain functional TNLs, whereas others do not. The same authors identified only 19 and 13 full length CNLs in the two Asteraceae, sunflower and lettuce, but no full length TNLs, and concluded that the latter are relatively poorly distributed or have been species (or family) specifically lost in Asterids. In line with our results, Christopoulu *et al*.^53^ recently identified several RGA classes in lettuce (one of the closest species to *C*. *cardunculus*), with the RLK class most represented. Other NBS-LRR like RGAs were found to vary widely in number (for a summary, see Sekhwal *et al*.^54^). Finally, it has been reported that NBS-LRR genes underwent gene expansion after speciation in *Arabidopsis* ^46, 55^, rice^56^, corn^57^, *Populus* and grape vine^58^. From our results, it appears that *C*. *cardunculus* RGAs belong almost exclusively to the RLK/RLP families, while a few NBS RGAs were identified. Our results confirm the hypothesis of Kim *et al*.^52^ of a species-specific evolution of TNLs in Asterids. Interestingly, several putative RGAs were identified and showed missing domains compared to the ‘canonical’ NBS, RLP and RLK families, in line with reports by several authors^59–68^. It was also reported that maintaining many NBS/resistance genes has potential fitness costs^69, 70^ and it has been suggested that plants use microRNAs to regulate NBS gene expression^71–75^. Indeed, we found that 9 (in VS) to 41 (in 2 C) identified RGAs (by Blastp approach) were putatively targeted by a miRNA, suggesting that this mechanism could also be present in *C*. *cardunculus*.

The number of identified miRNAs varied across the different genotypes, with the highest in 2 C and the lowest in VS. The variable number of identified miRNAs might also arise from SNPs present in some miRNA loci hampering their identification in some genotypes. In addition, as previously reported^23^, conserved miRNAs, such as miR162 and miR482, were not identified possibly because of their loss in the lines’ genomes and/or because of genomic loci missing in the assembly. Most of the conserved miRNAs detected in the six genomes were predicted to target well known biological processes; this confirms, on the basis of a more comprehensive data set, what has been previously reported^23, 76^. The comparison of GO term enrichments for the five newly reconstructed genomes revealed that GO terms related to binding and transcription were shared among the *C*. *cardunculus* genotypes, suggesting an involvement of miRNAs in transcription factors regulation. However, the process GO terms lignin and phenylpropanoids were enriched in 2 C, A41 and C3. This suggests their involvement in the regulation of genes involved in lignin biosynthesis and the flavonol pathway in these three genotypes, although this hypothesis should be furtherly investigated (e.g.: small RNA and degradome sequencing).

The comparison of the five reconstructed *C*. *cardunculus* sequences with the reference genome led to the discovery of more than 23 million SNP/indels, heterogeneously distributed across all the genotypes and their chromosomes revealing traces of breeding efforts. SNP frequency ranged from about 1/54 bp (VS) to 1/122 bp (A41), while indels frequency varied between about 1/630 bp (SP) to 1/1,634 bp (A41). In a previous study^77^, focused on RAD-tag marker development in globe artichoke, we estimated a SNP frequency of one SNP per 179 bp and one indel every 5000 bp, values which differ significantly from the those presented here (Table 4). This discrepancy may be attributed to the RAD-tag protocol we applied, which was based on the use of one methylation sensitive enzyme, thus the obtained metrics mainly refer to the un-transcribed portion of the genome.

As expected the globe artichoke reference genome (2 C) showed the lowest heterozygosity (0.11%, a frequency of 1/893 bp), which is comparable to that found in inbred species^78^. This reflects its breeding history as 2 C is the result of three cycles of self-fertilization. In contrast, according to Portis *et al*.^26, 79^, the four globe artichoke varietal types showed a high level of heterozygosity, as their heterozygous SNPs ranged from 7.4 M in VT to 9.3 M in VS (Table S11). Of these, the globe artichoke VT, the only genotype to be seed propagated, contained the lowest number of heterozygous SNPs (7,4 M); this is likely due to breeding practices aimed at decreasing its heterozygosity in order to stabilize its commercially-important attributes. Analogously, in the seed-propagated cultivated cardoon A41, a relatively low number (4,2 M) of heterozygous SNP was found. Many regions were observed with a low frequency of heterozygous SNPs (Fig. 3a) and carrying SNPs fixed in the homozygous state (Figure S6).

In a previous study, we identified Sicily Island, in the South of Italy, as a possible primary center of globe artichoke domestication. Following characterization of 24 landraces collected from small-holdings, through a combination of morphological traits and PCR-based markers, we recognized intermediary spiny forms in the domestication process^5^. The presence/absence of spines has been considered a key trait to understand the origin of the material at present in cultivation. Barbieri^80^ hypothesized that the spiny types (Spinosi) were selected first, followed by the violet types (Violetti), which possess less spiny heads, and finally by the non-spiny Romaneschi and Catanesi. However, both UPGMA analyses, performed on the whole set of detected SNPs/Indels and on the ones detected in coding regions, did not cluster separately the spiny and non-spiny types. This seems more consistent with the pattern of evolution proposed by Lanteri *et al*.^4^ according to which the Spinosi and the Violetti, the latter characterized by capitula with bracts harbouring fleshy thorns, evolved side by side with the non-spiny Catanesi and Romaneschi. Since domestication implies the intensification of pressure for traits relevant to farming conditions, and may have had a greater impact on genes than on intergenic regions, our hypothesis appears to be confirmed by the presence SNPs fixed in the homozygous state occurring in gene-dense regions in a genotype-specific fashion (Fig. 3a), which is attributable to a signature of the domestication process addressed to fix distinctive traits in the different varietal types.

The species *Cynara cardunculus* has interesting applications in pharmacology, since the leaves and heads represent natural sources of bio-active compounds such as mono- and di-caffeoylquinic acids and sesquiterpene lactones, with several medicinal properties. In previous studies we isolated, functionally characterized both ‘*in vitro*’ and *in vivo*’ and mapped the genes involved in their bio-synthetic pathway^81–84^. Our functional annotation of SNPs revealed thousands of coding polymorphisms (Table S12, Fig. 4) and the analysis of variants of genes related to caffeoylquinic acid (CQA) and sesquiterpene lactone (SL) biosynthesis displayed different outcomes. In the CQA pathway, except for one case, non-relevant deleterious SNPs were found. Conversely, in the SL pathway, although in heterozygosis, 54 deleterious mutations were found in 12 out of the 17 SL-related genes. This was confirmed by the remarkably lower diversity detected in CQA- compared to SL-related proteins (Fig. 5b). Many deleterious mutations were located in GAS, a key gene in the biosynthesis of SLs, of which cynaropicrin is the major representative in *C*. *cardunculus* ^83^. The latter was found to accumulate in leaf tissues while its concentration is lower in inflorescence bracts of globe artichokes^85, 86^; the same results were also confirmed in cultivated cardoon leaves^87^. Cynaropicrin acts in leaves as an antifeedant^88^ but is also responsible for the bitter taste of globe artichokes, thus the deleterious mutation in GAS gene detected in globe artichoke genotypes, but not in cultivated cardoon, might be the result of domestic breeding aimed at reducing bitterness in the globe artichoke edible capitula.

Next-generation sequencing is rapidly expanding our knowledge of genetic variation in many crops. The availability of the globe artichoke reference genome and the resequencing of four globe artichoke genotypes, representative of the germplasm in cultivation, has provided clues about their domestication processes as well as a first comprehensive identification of the genetic diversity of the *Cynara cardunculus* cultivated forms. For years, we have studied the progeny of the cross between the globe artichoke Romanesco with both globe artichoke and cultivated cardoon, with the goal to develop molecular maps on the basis of the two-way pseudo test cross strategy^24, 25^, and we have identified several QTLs for a number of capitula traits^26, 89, 90^. Based on our resequencing effort, a set of SNPs regularly spaced along the chromosomes was identified and annotated. This resource will be fruitful for the identification of regions responsible for the QTL, and lays the groundwork for a new phase of globe artichoke genomics to further the understanding of the genetic basis of agronomical important traits and for selective breeding. Beyond, our marker catalogue provides a highly valuable resource in terms of polymorphism, and allows foreseeing the future through the detection of fine haplotypes and imputation of SNPs on large accessions panels. Our results also provide key information for further functional, as well as comparative, genomics studies with other important crops such as sunflower, lettuce and chicory within the Asteraceae family.

## Materials and Methods

### Plant materials and DNA extraction

Six *C*. *cardunculus* genotypes (Table 1) were considered in the analysis; they included one cultivated cardoon (‘A41’ - Altilis 41), and five globe artichokes: ‘2 C’ (reference genome), VS (‘Violetto di Sicilia’), VT (‘Violetto di Toscana’), C3 (‘Romanesco C3’) and SP (‘Spinoso di Palermo’). SP belongs to the ‘*Spinosi’* type, characterized by long sharp spines on bracts and leaves. VT belongs to the ‘*Violetti’* type, produces medium-sized, green-violet-colored capitula harbouring fleshy thorns and it is seed-propagated. C3 belongs to the ‘*Romaneschi’* type, and its capitula are spherical, green and non-spiny. VS belongs to the ‘*Catanesi’* type, yielding non spiny elongated capitula with more or less marked violet streaks. 2 C is a Brazilian breeding line result of three cycles of selfing and characterized by a reduced level of heterozygosity^23^. A41 is a selected genotype of cultivated cardoon^25^ producing small non-spiny capitula and it is seed-propagated.

All of them derived from propagated clones grown in a field trial location at University of Catania (Italy), during the 2014–2015 growing season. For the genotypes VS, VT, SP, total genomic DNA was extracted from fresh leaves of each genotype, using DNA Mini Plant kit (Qiagen). RNAse A was used to remove RNA contamination. DNA quality was checked by 1% (w/v) agarose gel electrophoresis, and its quantity was assessed by Qubit 2.0 (Life Technologies, Carlsbad, CA, USA) based on Qubit dsDNA HS Assay (Life Science). Raw sequence data for the genotypes A41, 2 C, and C3 were obtained from NCBI (Table 2)^23^.

### Genome Sequencing

A total amount of one μg DNA was sonicated using 30″/90″ on/off time for 7 cycles with Bioruptor UCD-300 TS instrument (Diagenode, Belgium) to obtain 350 bp long fragments. End-repair and A-tailing procedures followed standard Illumina protocols, except PCR-free barcoded adapters (Biooscientific, Austin, TX, USA), used during the ligation step, and five conclusive enrichment PCR cycles were carried out. Negative selection of 100–150 bp fragments was performed with 0.8 X AMpure XP *beads* (Beckman Coulter, Inc., (Brea, California)). Quality control (QC) of libraries was performed with Bioanalyzer 2100 instrument (Agilent, Inc., Santa Clara, CA, USA) using High Sensitivity DNA kit and an accurate quantification was made using qPCR with Library Quantification kit (Kapa Biosystem, USA). Library were then pooled and diluted to a final concentration of 10 nM. Sequencing were performed with Illumina NextSeq500 sequencer (Illumina Inc., San Diego, CA, USA) and 150 bp paired-end sequences were generated. Raw reads were analyzed with Scythe (https://github.com/vsbuffalo/scythe) for filtering out contaminant substrings and Sickle (https://github.com/najoshi/sickle), which allows to remove reads with poor quality ends (Q < 30).

### Genome reconstruction, gene prediction and annotation

For the genome reconstruction, a combination of iterative read mapping against the globe artichoke reference and *de novo* assembly was adopted. *De novo* assembly was performed with ABySS 1.9.0 assembler^91^ using k-mers which allowed to achieve the best assembly. Genome reconstruction of each varietal type was performed submitting the Abyss assembled sequences to IMR/DENOM^41^ (ver. 0.4.1; http://mus.well.ox.ac.uk/) pipeline using default parameters, adopting the globe artichoke genome 2 C (LEKV00000000.1) as a guide. Quality metrics for the assembled genomes were calculated with Assemblathon_stats.pl (http://korflab.ucdavis.edu/). Gene prediction was performed using reiterative runs of the Maker-P suite^92^. HMM models from Augustus^93^ and SNAP^94^ *ab initio* gene prediction algorithms previously developed^23^ were combined with proteins and ESTs alignments as supporting evidence. All predicted gene models were filtered to retain only those with an AED ≤ 0.5. Gene function was assigned to predicted genes using BlastN^95^ and Swissprot^96^ database, using default parameters, with the exception of sequence E value = 1 e^−5^. Predicted protein sequences were functionally annotated using InterproScan^32^ (ver. 5.18–57.0) against all the available databases (ProSiteProfiles-20.119^97^, PANTHER-10.0^98^, Coils-2.2.1^99^, PIRSF-3.01^100^, Hamap-201511.02^101^, Pfam-29.0^102^, ProSitePatterns-20.119^97^, SUPERFAMILY-1.75^33^, ProDom-2006.1^103^, SMART-7.1^104^, Gene3D-3.5.0^105^ and TIGRFAM-15.0^106^). If InterProScan^32^ results were available, the domain name, IPR codes and GO terms^31^ were extracted and appended to the description line. The 6 sequences, together with their structural and functional annotation, are available through a JBrowse^28^ interface at http://www.artichokegenome.unito.it/jbrowse. The proteomes from the six genotypes were clustered using OrthoMCL^29^ version 2.0.9, a Venn diagram of the 6 proteomes was constructed with Interactivenn^107^ and AGRIGO^30^ cross comparison of SEA (SEACOMPARE) was used to identify common and different enrichment GO terms for specific gene clusters.

### Identification and characterization of PAV genes

Samtools^108^ was used to generate a text file containing the number of illumina reads that mapped at each gene location on the reference genome. The number of reads that mapped at each gene location for every globe artichoke varieties were normalized by the total number of reads mapping the whole reference genome for each four globe artichoke varieties. These figures were calculated as follows: *samtools view -c -F 4 -q 1 mapping_file*.*sorted*.*bam*. As approach to identify putative PAV genes, all genes with less than 6 mapped reads from at least 1 varietal type and more than 29 mapped reads from at least another varietal type were selected. A Venn diagram of the shared/exclusive PAV genes was depicted using InteractiVenn^107^. Obtained list of candidate PAV genes were described/GO-categorised using the here produced functional annotation and through a blastP^95^ analysis (TAIR10^109^). GO enrichments in artichoke selected genes were calculated with AmiGO2^110^ web service and Panther^111^.

### miRNA annotation

The MIReNA^112^ software was used for the identification of high confidence miRNA-coding sequences (miRBase release 21^34^: high confidence database) in each pseudomolecule and CH0 of all the six genotypes in study. An homology search was conducted with known miRNAs from an array of 13 species (plants and algae), including: *Solanum lycopersicum*, *Solanum tuberosum*, *Nicotiana tabacum*, *Vitis vinifera*, *Arabidopsis thaliana*, *Oryza sativa*, *Populus trichocarpa*, *Medicago trunculata*, *Zea mays*, *Picea abies*, *Triticum aestivum*, *Physcomitrella patens*, *Chlamydomonas reinhardtii*. MIReNA was run with default parameters and the maximum number of allowed mismatches between known miRNAs and putative miRNAs was set to 10. For each genotypes, miRNA sequences identified were named based on the miRNA family with the addition of the name of the genotype (2 C, A41, VS, VT, C3 and SP). The Tapir^35^ standalone software was applied to identify the targets of the identified eggplant miRNA on the predicted CDSs. In particular, we applied the Tapir hybrid function, which is based on the RNA hybrid search engine^113^, an algorithm conceived to determine, with high accuracy, miRNA:mRNA duplexes. Results were parsed with hybrid_parser function using default parameters. GO term enrichment of target sequences for each line was carried out with AGRIGO^30^ and REVIGO^36^ to find out a representative subset of the GO terms previously identified with the Interproscan^32^ pipeline (medium similarity) and to visualize results. The size of the circles have been adjusted to reflect the p-value. AGRIGO^30^ cross comparison of SEA (SEACOMPARE) was used to identify common and different enrichment GO terms between the genotypes showing GO terms enrichment.

### Resistance genes analogs (RGA) identification and classification

Candidates genes were identified by means of a Blastp^95^ analysis against the Plant Resistance Genes database^114^ (http://prgdb.crg.eu/). Protein sequences of 2860 *Arabidopsis* RGAs (from RGDB) were used to perform BlastP searches against all the six proteomes. For each genotype, positive hits (p < 1e-60) were validated via HMMER37,38 v3 software, searching against PFAM^102^ Hidden Markov Models (HMMs: available at http://pfam.xfam.org/) using a cutoff E-value of 1e-10. HMMs domains were chosen based on their known involvement in plant resistance against pathogens and included: NB-ARC (PF00931), TIR (PF01582), LRR (PF00560, PF07723, PF007725, PF08263, PF12799, PF13306, PF13516, PF13855 and PF14580), Pkinase (PF00069), ABC transporter (PF00005), and WD40 (PF00400). The CC motifs were predicted with EMBOSS pepcoil^115^, while TM domains were predicted using both TMHMM 2.0^116^ and SCAMPI^117^. Resistance genes classification was based as followed: acronym containing the same domains from Christoupolou *et al*.^53^ were used. For missing domains combination, the acronyms were generated by adding to ‘other’ the following letters: L (LRR), N (NB-ARC), TM (TM), T (TIR), C (CC), K (Pkinase), and A (ABC transporter).

### RGAs alignment and phylogenetic tree estimation

To identify the RGAs evolution within and among the 6 lines in study, we divided the RGAs identified following Hmmer analyses in 6 separated groups: RLP (Receptor Like Proteins), RLK (Receptor like Kinases), NB (proteins containing at least one NB-ARC domain), LRR (containing only LRR domains), ABC (containing at least one ABC domain) and other-KTM (containing a Kinase and a TM domains and not belonging to RLK/RLP group). The multiple alignments were performed using MAFFT^118^ v7.294b with the following parameters:–ep 0–thread 22–reorder–maxiterate 1000–genafpair. Phylogenetic trees were inferred with RAxML^39^ v.8.2.8 using the Maximum Likelihood method based on the GAMMAPROTILGF method (LG matrix with GAMMA model of heterogeneity, empirical amino acid frequency and estimate of proportion of invariable site). The tree with the highest log-likelihood was selected and combined with the bootstrap output to print support values assigned to nodes. For bootstrap analyses, RAxML^39^ v.8.2.8 was used, with fast bootstrapping^119^ and outMRE option. FigTree 1.4.2^120^ was used to display all the six dendrograms in cladogram format. To identify the number of RGAs per chromosome across all the 6 genotypes, as well as the presence of clusters, coordinates of the genes belonging to the classes RLK, RLP, NB (containing at least a NB domain), and other-KTM were extracted and BEDTools^121^ intersected using genome windows of 1 Mb to count the number of genes falling into these regions.

### SNP calling

Reads were mapped onto globe artichoke genome reference using Burrows-Wheeler Aligner program (BWA)^122^ and ‘mem’ command with default parameters. The BAM files were processed and adapted for SNP calling program with Samtools^108^ mpileup using default parameters with the exclusion of minimum mapping quality equal to 25 and filtering ambiguous read mapping. Results were filtered taking into account two parameters: the SNPs call quality and depth. SNPs having mapping quality lower than 20 were removed. In addition, we set as lower limit of mapping depth a value of eight and the upper limit was set to 450. Relationships among the genotypes were computed using: i) whole genome and ii) coding (within exons) SNP/indel datasets. Genetic distances were computed based on the two datasets (R package SNPRelate) and dendrogram was computed and drawn using R Graphic-package^123^. The chromosomal locations of SNP densities were visualized in CIRCOS ideograms using the software package from http://circos.ca.

### SNP annotation

Identified variants were analyzed using SNPeff^40^ to predict their effect on the set of gene models of globe artichoke. We investigated the role of missense and non-sense mutations in both homozygous/heterozygous states, evaluating in which region the variations are found. The effect of each SNP/indel was classified according to SNPeff software into four classes: (1)”modifier”, for the variants located outside the genes, in non-transcribed regions or introns; (2) “low effect” for variants in coding regions which do not change the amino acid sequence; (3) “moderate” effect for variants which change the amino acid sequence and (4) “high effect” for variants which modify splice sites, stop or start codons (loss or gain). CDS non-synonymous variants belonging to well characterized pathway and classified as missense, stop codon gained and frameshift effect were also submitted to Provean (Protein Variation Effect Analyzer algorithm)^124^ analysis, in order to understand if an amino acid substitution has an impact on the biological protein functions. Provean predicts the functional impact for all classes of protein sequence variation such a single amino acid substitution, insertion, deletion and multiple substitution. The score threshold used was set to −2.5. Non-synonymous variations causing deleterious effects are evaluated in homozygous and heterozygous state for the negative impact on protein functionality.

### Accession codes

Sequence reads have been deposited in NCBI sequence read archive (SRA) under the number SRP055806. A JBrowse^28^ interface, to access genomic data and related annotation, is available at www.artichokegenome.unito.it.

## Electronic supplementary material

Supplementary File S1

Supplementary File S2

Supplementary File S3

Supplementary File S4

Supplementary informations and file S5
