# Supplementary material for: Genome reconstruction in Cynara cardunculus taxa gains access to chromosome-scale DNA variation
Source: Sci Rep. 2017 Jul 17;7:5617. doi: 10.1038/s41598-017-05085-7 (PMC5514137; doi:10.1038/s41598-017-05085-7)
Supplement: Supplementary file 5 — Supplementary informations and file S5 [file 41598_2017_5085_MOESM5_ESM.pdf]

Genome reconstruction in *Cynara cardunculus* taxa gains access to chromosome-scale DNA variation

Alberto Acquadro[1], Lorenzo Barchi[1], Ezio Portis[1,*], Giulio Mangino[1], Danila Valentino[1], Giovanni Mauromicale[2], Sergio Lanteri[1]

Addresses

[1] DISAFA, Plant Genetics and Breeding, University of Torino, Grugliasco, Italy.
[2] Dipartimento di Agricoltura, Alimentazione e Ambiente (Di3A), University of Catania, Catania, Italy.
[*] Corresponding author.

Inventory of Supplementary Information:

- Supplementary Figures S1-S6
- Supplementary Tables S1-S14
- Supplementary data
- Supplementary file list (S1, S2, S3, S4, S5, S6)

Supplementary FIGUREs

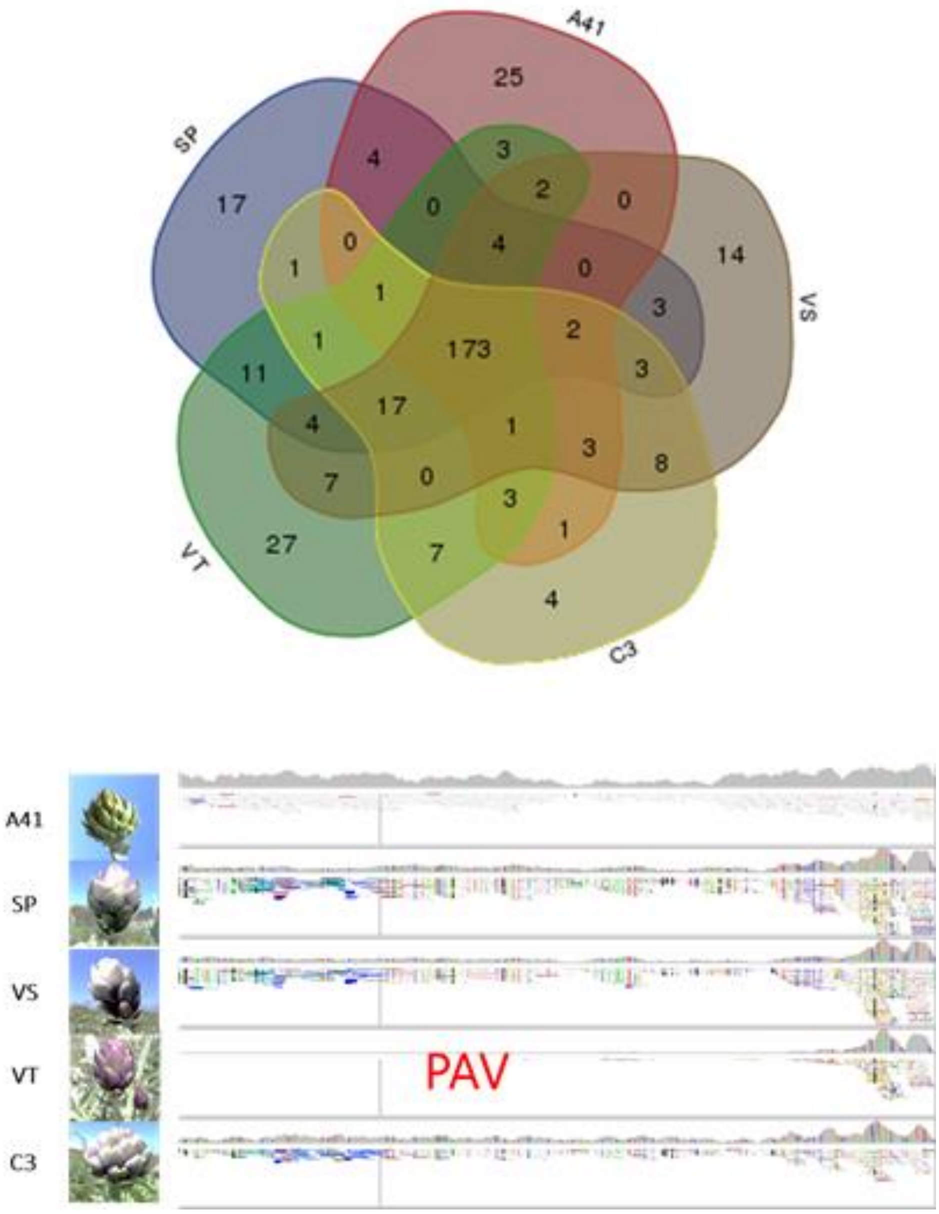

Supplementary Figure S1 | PAV categorization. Top part) Diagram shows the number of genes putatively absent in the resequenced genotypes compared to reference (2C). Bottom part) A detail of a PAV region (absent for the VT genotype).

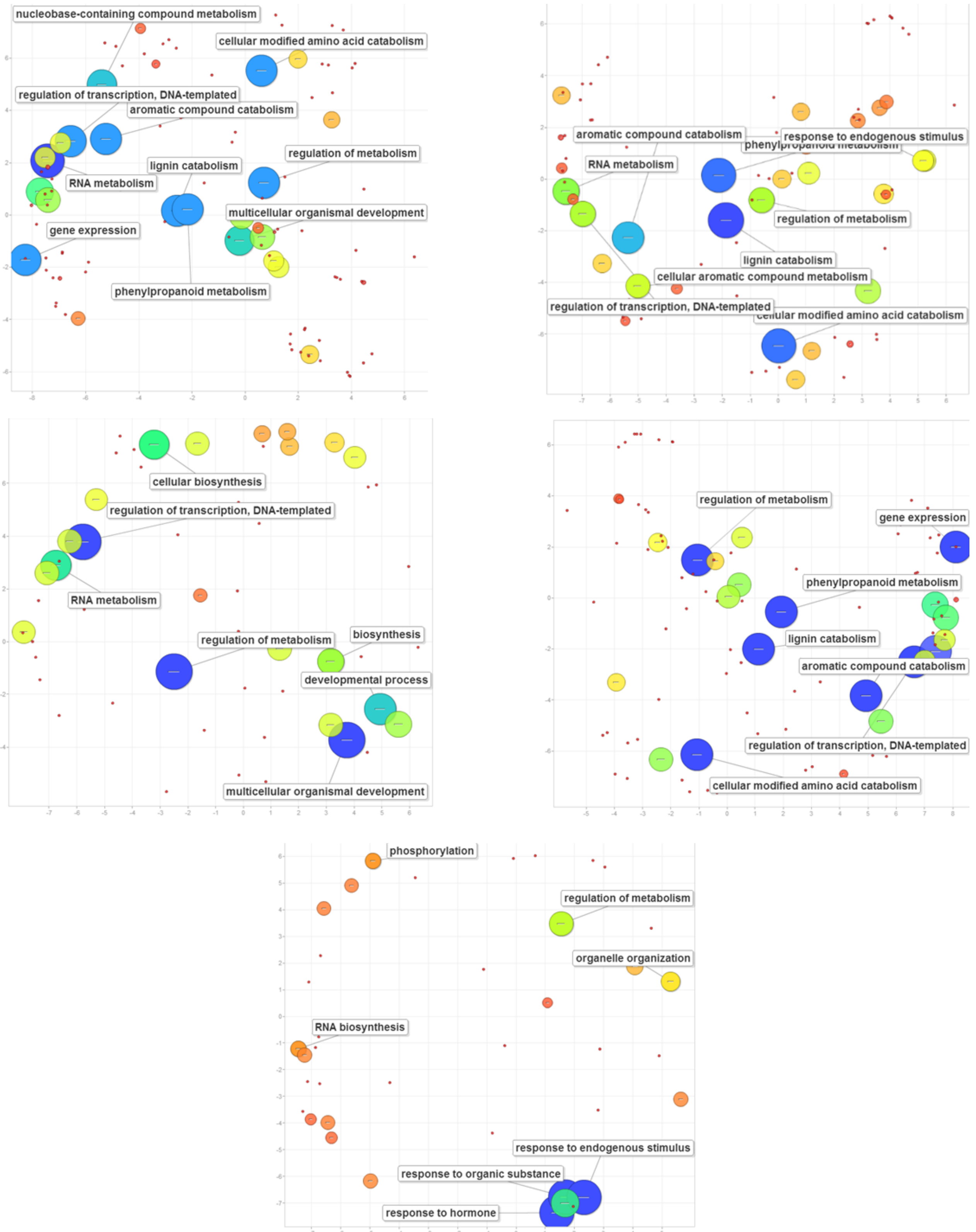

Supplementary Figure S2 | REVIGO[1] summarization of enriched terms for biological process of miRNAs target genes in Reference (top left), A41 (top right), VS (middle left), C3 (middle right) and SP (bottom). Bubbles size is proportional of log10-pvalue of enrichment analyses; bubbles color is also in function of log10-pvalue of enrichment analyses (blue low-red-high p-value). X and Y axes represent semantic similarity according to Revigo algorithm (similar GO terms remains close together).

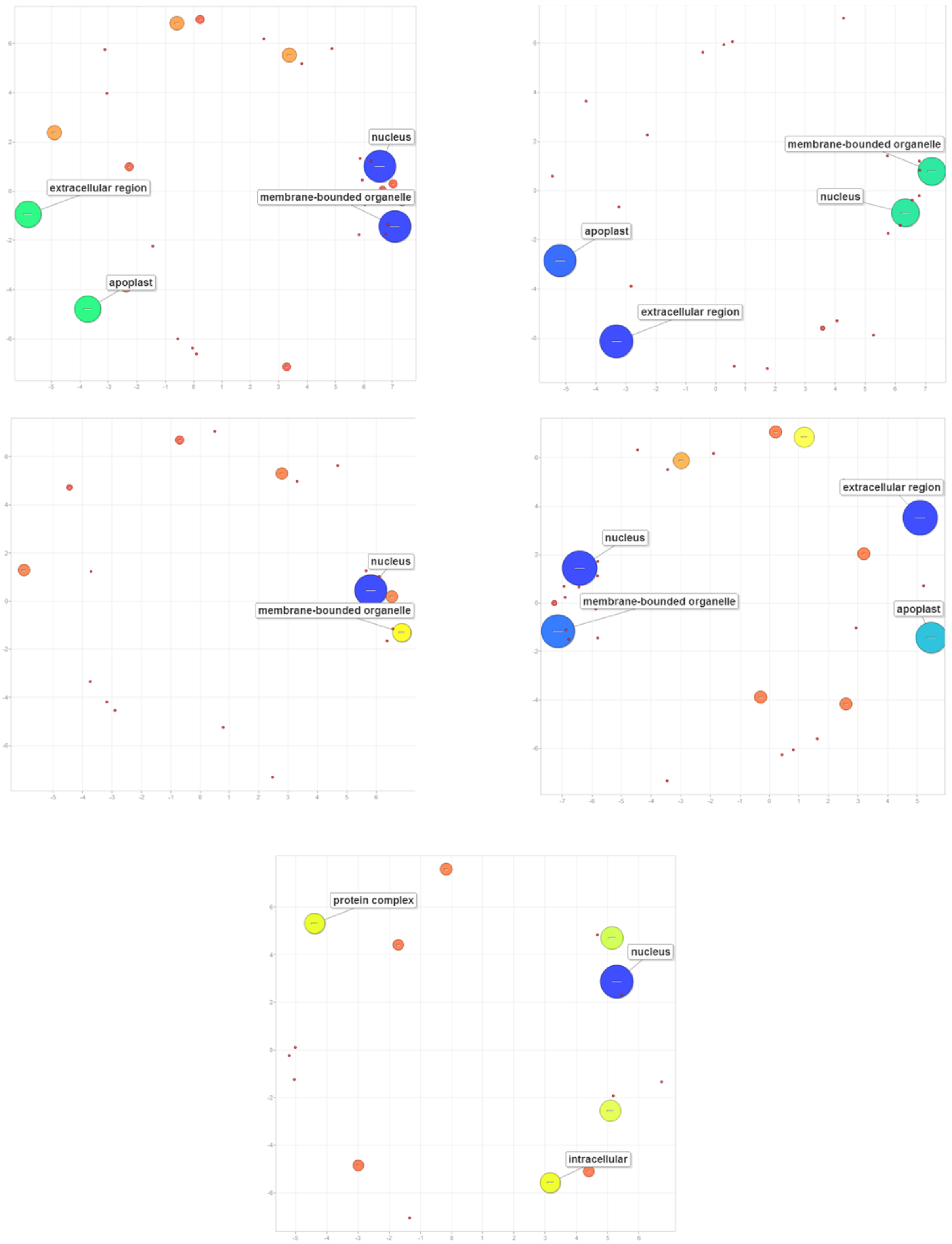

Supplementary Figure S3 | REVIGO[1] summarization of enriched terms for cellular component of miRNAs target genes in Reference (top left), A41 (top right), VS (middle left), C3 (middle right) and SP (bottom). Bubbles size is proportional of log10-pvalue of enrichment analyses; bubbles color is also in function of log10-pvalue of enrichment analyses (blue low-red-high p-value). X and Y axes represent semantic similarity according to Revigo[32] algorithm (similar GO terms remains close together).

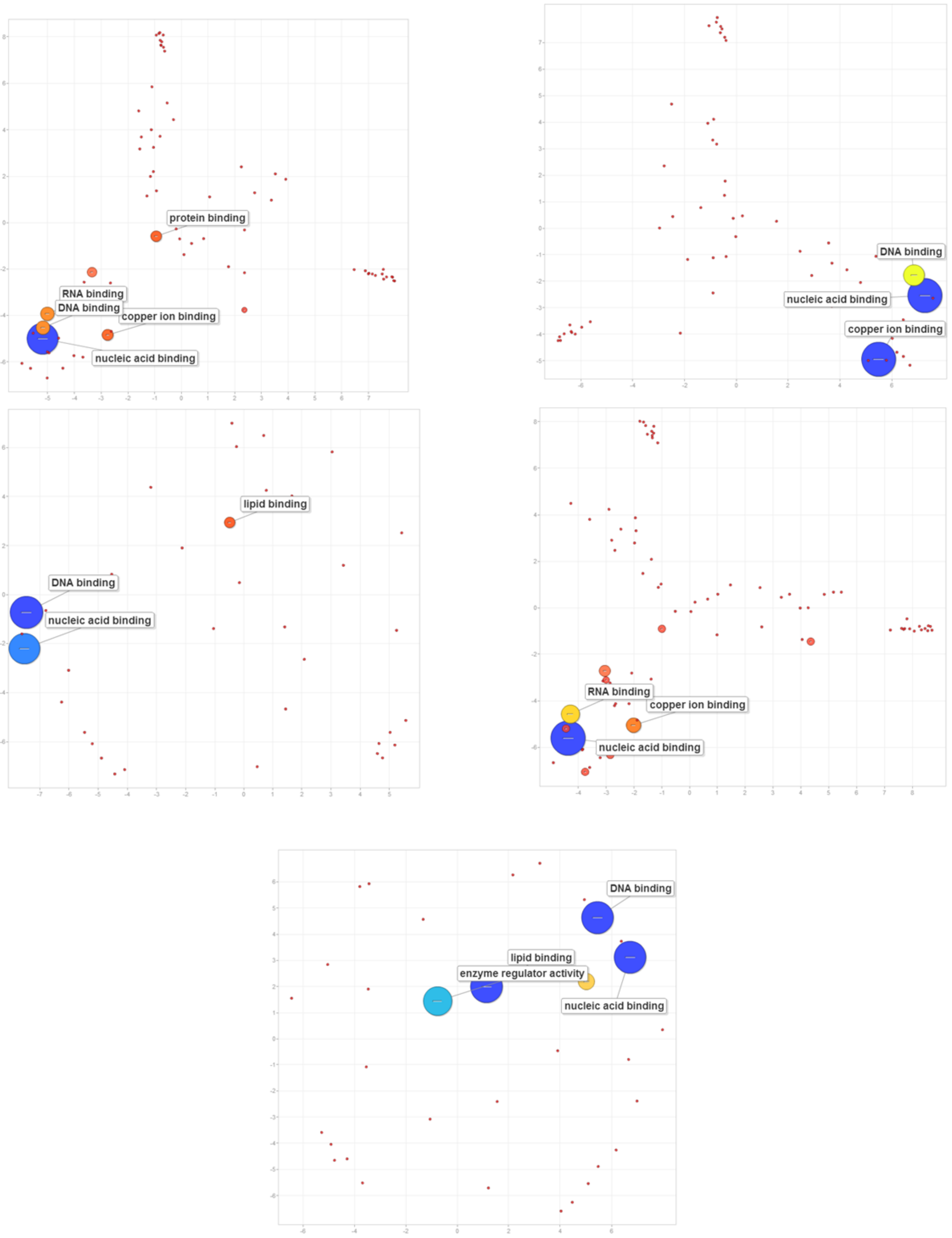

Supplementary Figure S4 | REVIGO[1] summarization of enriched terms for molecular function of miRNAs target genes in Reference (top left), A41 (top right), VS (middle left), C3 (middle right) and SP (bottom). Bubbles size is proportional of log10-pvalue of enrichment analyses; bubbles color is also in function of log10-pvalue of enrichment analyses (blue low-red-high p-value). X and Y axes represent semantic similarity according to Revigo[32] algorithm (similar GO terms remains close together).

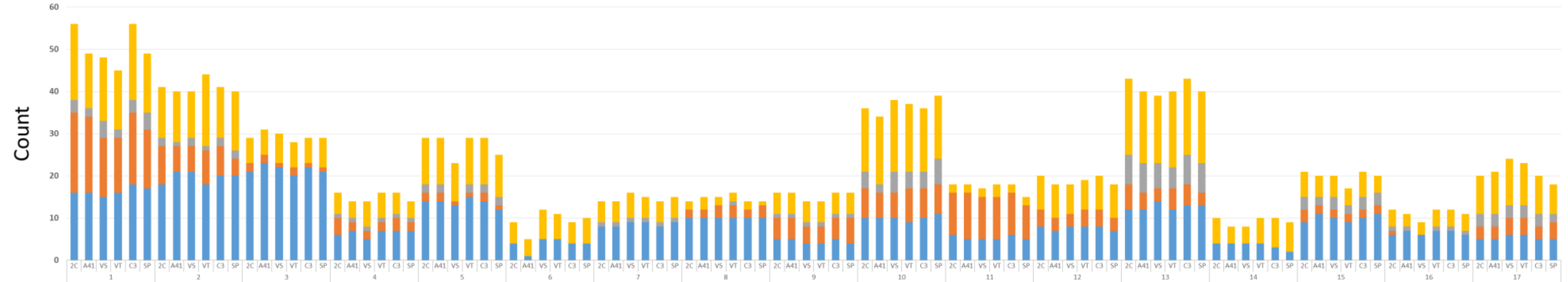

Chromosome	Genotype	otherKTM class	NB class	RLP class	RLK class
1	2C	18	3	19	16
1	A41	13	2	18	16
1	VS	15	4	14	15
1	VT	14	2	13	16
1	C3	18	3	17	18
1	SP	14	4	14	17
2	2C	12	2	9	18
2	A41	12	1	6	21
2	VS	11	2	6	21
2	VT	17	1	8	18
2	C3	12	2	7	20
2	SP	14	2	4	20
3	2C	6	0	2	21
3	A41	6	0	2	23
3	VS	7	0	1	22
3	VT	6	0	2	20
3	C3	6	0	1	22
3	SP	7	0	1	21
4	2C	5	1	4	6
4	A41	4	1	2	7
4	VS	6	1	2	5
4	VT	6	1	2	7
4	C3	5	1	3	7
4	SP	4	1	2	7
5	2C	11	2	2	14
5	A41	11	2	2	14
5	VS	9	0	1	13
5	VT	11	2	1	15
5	C3	11	2	2	14
5	SP	10	2	1	12
6	2C	5	0	0	4
6	A41	4	0	0	1
6	VS	7	0	0	5
6	VT	6	0	0	5
6	C3	5	0	0	4
6	SP	6	0	0	4
7	2C	5	1	0	8
7	A41	5	1	0	8
7	VS	6	1	0	9
7	VT	5	1	0	9
7	C3	5	1	0	8
7	SP	5	1	0	9
8	2C	2	0	2	10
8	A41	3	0	2	10
8	VS	2	0	3	10
8	VT	2	1	3	10
8	C3	2	0	2	10
8	SP	1	0	3	10
9	2C	5	1	5	5
9	A41	5	1	5	5
9	VS	5	1	4	4
9	VT	5	1	4	4
9	C3	5	1	5	5
9	SP	5	1	6	4
10	2C	15	4	7	10
10	A41	16	2	6	10
10	VS	17	5	6	10
10	VT	16	4	8	9
10	C3	15	4	7	10
10	SP	15	6	7	11
11	2C	2	0	10	6
11	A41	2	0	11	5
11	VS	2	0	10	5
11	VT	3	0	10	5
11	C3	2	0	10	6
11	SP	2	0	8	5
12	2C	8	0	4	8
12	A41	8	0	3	7
12	VS	7	0	3	8
12	VT	7	0	4	8
12	C3	8	0	4	8
12	SP	8	0	3	7
13	2C	18	7	6	12
13	A41	17	7	4	12
13	VS	16	6	3	14
13	VT	18	5	5	12
13	C3	18	7	5	13
13	SP	17	7	3	13
14	2C	6	0	0	4
14	A41	4	0	0	4
14	VS	4	0	0	4
14	VT	6	0	0	4
14	C3	7	0	0	3
14	SP	7	0	0	2
15	2C	6	3	3	9
15	A41	5	2	2	11
15	VS	5	3	2	10
15	VT	4	2	2	9
15	C3	6	3	2	10
15	SP	4	3	2	11
16	2C	4	1	1	6
16	A41	3	1	0	7
16	VS	3	0	0	6
16	VT	4	1	0	7
16	C3	4	1	0	7
16	SP	4	1	0	6
17	2C	9	3	3	5
17	A41	10	3	3	5
17	VS	11	3	4	6
17	VT	10	3	4	6
17	C3	9	3	3	5
17	SP	7	2	4	5

Supplementary Figure S5 | Distribution of RLK, RLP, NB and other-KTM RGAs. Gene distribution for each class is shown across the 17 C. cardunculus chromosomes for each of the 6 genotypes. Bars are divided into RLK genes (blue), RLPs (orange), NB (grey) and other-KTM (yellow).

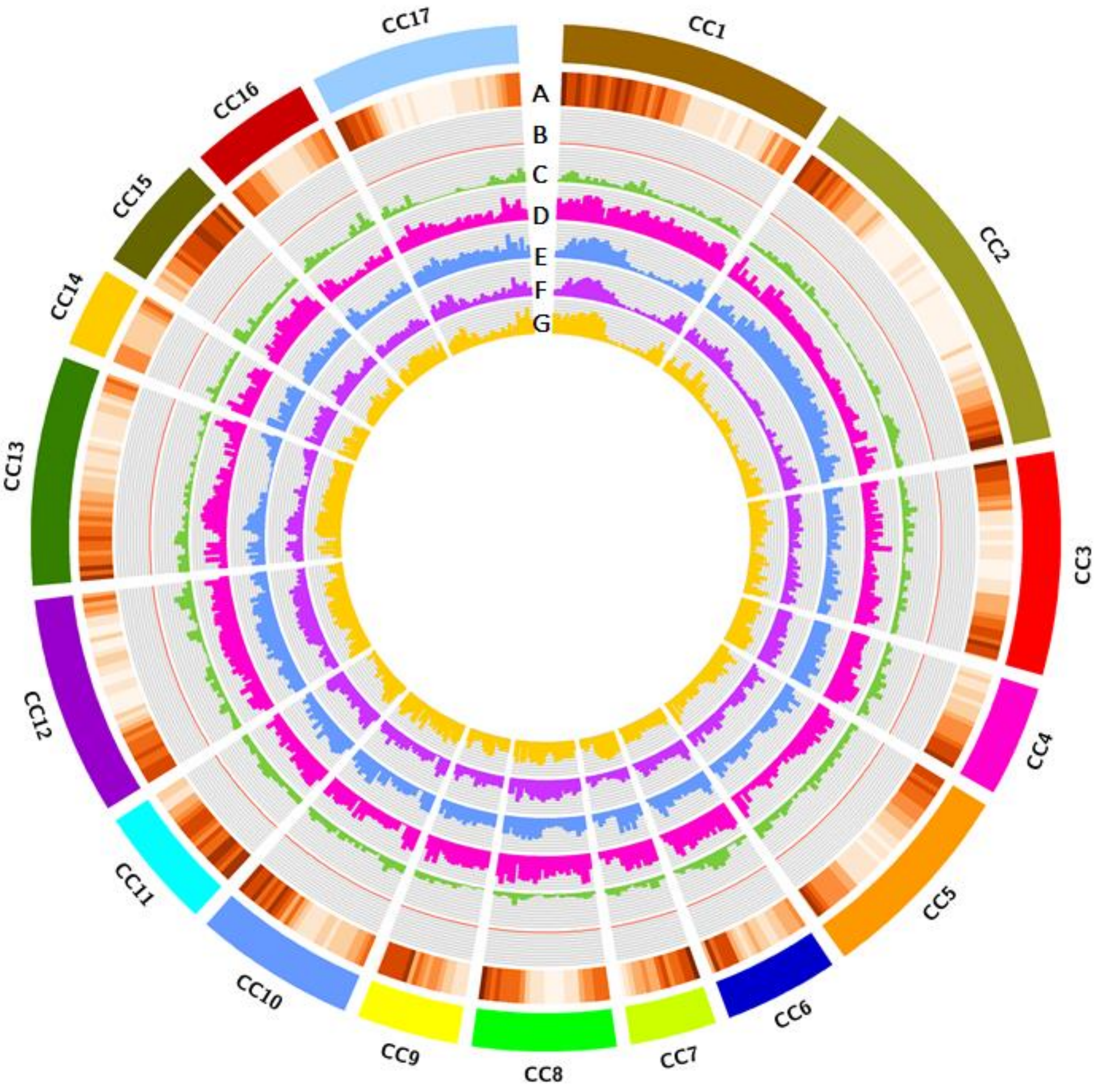

Supplementary Figure S6 | Circos diagram depicting gene and homozygous SNP densities; from the outer circle to the inner circle: A) Heat map of gene density in the reference genome; 1M histograms representing the density of heterozygous SNPs for the Reference (track B) A41 (track C), SP (track D), VS (track E), C3 (track F), VT (track G).

Supplementary Tables

Supplementary Table S1 | Distribution of orthologous gene families in the six *C. Cardunculus* genotypes using OrthoMCL[2]. The genomes shared gene families were depicted as cells highlighted in colors.

	REF	A41	VS	VT	C3	SP	No. of gene families	No. of genes within gene family
CORE							18826	138098
FIVE							362	1836
							68	354
							380	1932
							438	2216
							426	2145
							15	77
FOUR							16	65
							179	719
							23	96
							214	861
							22	88
							221	886
							90	361
							10	41
							89	360
							103	415
							2	8
							99	403
							4	17
							6	24
							26	104
THREE							19	57
							40	121
							475	1427
							28	85
							6	18
							88	264
							5	15
							118	354
							9	28
							123	369
							33	101
							9	27
							38	114
							4	12
							48	147
							4	12
							12	36
							149	449
							14	42
							16	48
TWO							105	215
							14	29
							27	55
							592	1188
							19	39
							99	200
							62	124
							12	24
							69	149
							159	318
							19	38
							148	296
							22	44
							174	348
							26	52
UNIQUE							11	24
							1	2
							1	2
Genes in cluster	28161	26786	26250	26384	27840	26434		
Singletons	149	999	871	776	189	892		

Supplementary Table S2 | SEACOMPARE from AGRIGO[3] of the GO terms enriched for genotypic exclusive genes

GO Information				CM					2C		A41		VS		C3		SP	
No	GOTerm	Onto	Description	2C	A41	VS	C3	SP	FDR	Num	FDR	Num	FDR	Num	FDR	Num	FDR	Num
1	GO:0016070	P	RNA metabolic process						0.017	95	0.025	63	0.028	33	0.025	90	---	---
2	GO:0080090	P	regulation of primary metabolic process						0.03	65	0.033	46	0.0065	29	0.025	63	---	---
3	GO:0019222	P	regulation of metabolic process						0.03	67	0.033	47	0.0065	30	0.025	65	---	---
4	GO:0009698	P	phenylpropanoid metabolic process						0.03	6	0.002	6	---	---	0.025	6	---	---
5	GO:0060255	P	regulation of macromolecule metabolic process						0.03	66	0.033	46	0.0065	29	0.025	64	---	---
6	GO:0019439	P	aromatic compound catabolic process						0.03	7	0.0047	6	---	---	0.025	7	---	---
7	GO:0009889	P	regulation of biosynthetic process						0.03	63	0.033	44	0.0065	27	0.025	61	---	---
8	GO:0006355	P	regulation of transcription, DNA-dependent						0.03	63	0.033	44	0.0065	27	0.025	61	---	---
9	GO:0006350	P	transcription						0.03	72	0.033	50	0.0065	30	0.03	68	---	---
10	GO:0006351	P	transcription, DNA-dependent						0.03	72	0.033	50	0.0065	30	0.03	68	---	---
11	GO:0032774	P	RNA biosynthetic process						0.03	72	0.033	50	0.0065	30	0.03	68	---	---
12	GO:0009808	P	lignin metabolic process						0.03	6	0.0013	6	---	---	0.025	6	---	---
13	GO:0046274	P	lignin catabolic process						0.03	6	0.0013	6	---	---	0.025	6	---	---
14	GO:0046271	P	phenylpropanoid catabolic process						0.03	6	0.0013	6	---	---	0.025	6	---	---
15	GO:0051252	P	regulation of RNA metabolic process						0.03	63	0.033	44	0.0065	27	0.025	61	---	---
16	GO:0031326	P	regulation of cellular biosynthetic process						0.03	63	0.033	44	0.0065	27	0.025	61	---	---
17	GO:0045449	P	regulation of transcription						0.03	63	0.033	44	0.0065	27	0.025	61	---	---
18	GO:0042219	P	cellular amino acid derivative catabolic process						0.03	6	0.002	6	---	---	0.025	6	---	---
19	GO:0010467	P	gene expression						0.03	112	---	---	---	---	0.025	107	---	---
20	GO:0010556	P	regulation of macromolecule biosynthetic process						0.03	63	0.033	44	0.0065	27	0.025	61	---	---
21	GO:0010468	P	regulation of gene expression						0.03	64	0.043	44	0.0083	27	0.025	62	---	---
22	GO:0019219	P	regulation of nucleobase, nucleoside, nucleotide and nucleic acid metabolic process						0.03	63	0.033	44	0.0065	27	0.025	61	---	---
23	GO:0051171	P	regulation of nitrogen compound metabolic process						0.03	63	0.033	44	0.0065	27	0.025	61	---	---
24	GO:0006139	P	nucleobase, nucleoside, nucleotide and nucleic acid metabolic process						0.041	107	---	---	---	---	---	---	---	---
25	GO:0031323	P	regulation of cellular metabolic process						0.047	64	0.042	45	0.0065	28	0.037	62	---	---
26	GO:0007275	P	multicellular organismal development						0.047	10	---	---	0.0065	7	---	---	---	---
27	GO:0003676	F	nucleic acid binding						0.0034	153	0.034	100	0.0049	58	0.011	145	---	---
28	GO:0043227	C	membrane-bounded organelle						0.019	72	0.012	50	---	---	0.035	67	0.049	25
29	GO:0043231	C	intracellular membrane-bounded organelle						0.019	72	0.012	50	---	---	0.035	67	0.049	25
30	GO:0005634	C	nucleus						0.019	53	0.012	36	0.019	22	0.026	49	0.0018	23
31	GO:0019748	P	secondary metabolic process						---	---	0.033	7	---	---	---	---	---	---
32	GO:0006725	P	cellular aromatic compound metabolic process						---	---	0.048	10	---	---	---	---	---	---
33	GO:0005507	F	copper ion binding						---	---	0.034	9	---	---	---	---	---	---
34	GO:0005576	C	extracellular region						---	---	0.002	10	---	---	0.026	11	---	---
35	GO:0048046	C	apoplast						---	---	0.0028	7	---	---	---	---	---	---
36	GO:0032502	P	developmental process						---	---	---	---	0.021	7	---	---	---	---
37	GO:0044249	P	cellular biosynthetic process						---	---	---	---	0.034	49	---	---	---	---
38	GO:0003677	F	DNA binding						---	---	---	---	0.0027	42	---	---	---	---
39	GO:0009719	P	response to endogenous stimulus						---	---	---	---	---	---	---	---	0.045	7
40	GO:0010033	P	response to organic substance						---	---	---	---	---	---	---	---	0.045	7
41	GO:0009725	P	response to hormone stimulus						---	---	---	---	---	---	---	---	0.045	7

Supplementary Table S3 | TOP20 Superfamily[4] for each of the 6 genotypes, after Interproscan[5] analyses.

2C

Domain	DESCRIPTION	Count
SSF52540	P-loop containing nucleoside triphosphate hydrolase	1604
SSF56112	Protein kinase-like domain	1437
SSF52058	Leucine-rich repeat domain, L domain-like	978
SSF48371	Armadillo-type fold	905
SSF48452	Tetratricopeptide-like helical domain	558
SSF51735	NAD(P)-binding domain	475
SSF53474	Alpha/Beta hydrolase fold	465
SSF54928	Nucleotide-binding alpha-beta plait domain	445
SSF50978	WD40-repeat-containing domain	445
SSF48264	Cytochrome P450	386
SSF46689	Homeodomain-like	368
SSF53335	S-adenosyl-L-methionine-dependent methyltransferase	345
SSF103473	Major facilitator superfamily domain	329
SSF51905	FAD/NAD(P)-binding domain	290
SSF52833	Thioredoxin-like fold	276
SSF51445	Glycoside hydrolase superfamily	271
SSF49503	Cupredoxin	243
SSF81383	F-box domain	242
SSF47473	EF-hand domain pair	238
SSF54171	DNA-binding domain	221

VS

Domain	DESCRIPTION	Count
SSF52540	P-loop containing nucleoside triphosphate hydrolase	1542
SSF56112	Protein kinase-like domain	1366
SSF52058	Leucine-rich repeat domain, L domain-like	917
SSF48371	Armadillo-type fold	876
SSF48452	Tetratricopeptide-like helical domain	537
SSF51735	NAD(P)-binding domain	457
SSF53474	Alpha/Beta hydrolase fold	449
SSF54928	Nucleotide-binding alpha-beta plait domain	430
SSF50978	WD40-repeat-containing domain	422
SSF48264	Cytochrome P450	365
SSF46689	Homeodomain-like	362
SSF53335	S-adenosyl-L-methionine-dependent methyltransferase	333
SSF103473	Major facilitator superfamily domain	318
SSF51905	FAD/NAD(P)-binding domain	288
SSF52833	Thioredoxin-like fold	275
SSF51445	Glycoside hydrolase superfamily	263
SSF81383	F-box domain	245
SSF49503	Cupredoxin	245
SSF47473	EF-hand domain pair	222
SSF56784	HAD-like domain	218

A41

Domain	DESCRIPTION	Count
SSF52540	P-loop containing nucleoside triphosphate hydrolase	1564
SSF56112	Protein kinase-like domain	1408
SSF52058	Leucine-rich repeat domain, L domain-like	927
SSF48371	Armadillo-type fold	890
SSF48452	Tetratricopeptide-like helical domain	542
SSF53474	Alpha/Beta hydrolase fold	457
SSF51735	NAD(P)-binding domain	454
SSF54928	Nucleotide-binding alpha-beta plait domain	441
SSF50978	WD40-repeat-containing domain	429
SSF48264	Cytochrome P450	384
SSF46689	Homeodomain-like	364
SSF53335	S-adenosyl-L-methionine-dependent methyltransferase	332
SSF103473	Major facilitator superfamily domain	331
SSF51905	FAD/NAD(P)-binding domain	291
SSF52833	Thioredoxin-like fold	274
SSF51445	Glycoside hydrolase superfamily	265
SSF49503	Cupredoxin	238
SSF81383	F-box domain	235
SSF47473	EF-hand domain pair	229
SSF56784	HAD-like domain	223

VT

Domain	DESCRIPTION	Count
SSF52540	P-loop containing nucleoside triphosphate hydrolase	1542
SSF56112	Protein kinase-like domain	1383
SSF48371	Armadillo-type fold	873
SSF52058	Leucine-rich repeat domain, L domain-like	863
SSF48452	Tetratricopeptide-like helical domain	534
SSF51735	NAD(P)-binding domain	451
SSF53474	Alpha/Beta hydrolase fold	450
SSF50978	WD40-repeat-containing domain	444
SSF54928	Nucleotide-binding alpha-beta plait domain	428
SSF48264	Cytochrome P450	364
SSF46689	Homeodomain-like	361
SSF53335	S-adenosyl-L-methionine-dependent methyltransferase	336
SSF103473	Major facilitator superfamily domain	322
SSF51905	FAD/NAD(P)-binding domain	291
SSF52833	Thioredoxin-like fold	272
SSF51445	Glycoside hydrolase superfamily	259
SSF49503	Cupredoxin	241
SSF81383	F-box domain	236
SSF47473	EF-hand domain pair	218
SSF56784	HAD-like domain	216

C3

Domain	DESCRIPTION	Count
SSF52540	P-loop containing nucleoside triphosphate hydrolase	1591
SSF56112	Protein kinase-like domain	1425
SSF52058	Leucine-rich repeat domain, L domain-like	954
SSF48371	Armadillo-type fold	905
SSF48452	Tetratricopeptide-like helical domain	563
SSF51735	NAD(P)-binding domain	464
SSF53474	Alpha/Beta hydrolase fold	462
SSF54928	Nucleotide-binding alpha-beta plait domain	446
SSF50978	WD40-repeat-containing domain	441
SSF48264	Cytochrome P450	378
SSF46689	Homeodomain-like	367
SSF53335	S-adenosyl-L-methionine-dependent methyltransferase	346
SSF103473	Major facilitator superfamily domain	327
SSF51905	FAD/NAD(P)-binding domain	290
SSF52833	Thioredoxin-like fold	274
SSF51445	Glycoside hydrolase superfamily	265
SSF49503	Cupredoxin	244
SSF81383	F-box domain	242
SSF47473	EF-hand domain pair	237
SSF54171	DNA-binding domain	221

SP

Domain	DESCRIPTION	Count
SSF52540	P-loop containing nucleoside triphosphate hydrolase	1544
SSF56112	Protein kinase-like domain	1368
SSF48371	Armadillo-type fold	893
SSF52058	Leucine-rich repeat domain, L domain-like	883
SSF48452	Tetratricopeptide-like helical domain	535
SSF51735	NAD(P)-binding domain	464
SSF53474	Alpha/Beta hydrolase fold	452
SSF50978	WD40-repeat-containing domain	440
SSF54928	Nucleotide-binding alpha-beta plait domain	431
SSF46689	Homeodomain-like	364
SSF48264	Cytochrome P450	356
SSF53335	S-adenosyl-L-methionine-dependent methyltransferase	335
SSF103473	Major facilitator superfamily domain	326
SSF51905	FAD/NAD(P)-binding domain	288
SSF52833	Thioredoxin-like fold	271
SSF51445	Glycoside hydrolase superfamily	259
SSF81383	F-box domain	241
SSF49503	Cupredoxin	240
SSF47473	EF-hand domain pair	228
SSF54171	DNA-binding domain	220

Supplementary Table S4 | List of putative PAV genes specifically absent in one of the resequenced accessions. Table shows the corresponding genes found in Arabidopsis as a result of a BlastX analysis on TAIR10[6].

Presence /Absence	Genotype	2C ortholog	Annotation	AGI homolog	GO
+	**A41**	Ccrd_v2_00760_g01	Similar to Probable histone H2B.1 (Medicago truncatula)	AT3G53650.1	GO:0046982,GO:0003677,GO:0005634
+		Ccrd_v2_02659_g01	Similar to At1g30570 Probable receptor-like protein kinase At1g30570 (Arabidopsis thaliana)	AT1G30570.1	GO:0009826,GO:0016301,GO:0006468,GO:0005524,GO:0009741,GO:0004674,GO:0005886,GO:0016301,GO:0016021,GO:0006468
+		Ccrd_v2_03517_g02	Protein of unknown function	AT1G73990.1	GO:0009534,GO:0009642,GO:0009535,GO:0006465,GO:0004252,GO:0009507,GO:0009941,GO:0016021,GO:0006508,GO:0006508,GO:0009507,GO:0005886,GO:0009570
+		Ccrd_v2_07693_g04	Similar to SBT1.7 Subtilisin-like protease SBT1.7 (Arabidopsis thaliana)	AT5G67090.1	GO:0005618,GO:0006508,GO:0008152,GO:0005618,GO:0005576,GO:0004252,GO:0005576,GO:0006508,GO:0004252
+		Ccrd_v2_12555_g08	Similar to Limonoid UDP-glucosyltransferase (Citrus unshiu)	AT4G15480.1	GO:0080043,GO:0008194,GO:0035251,GO:0050284,GO:0009636,GO:0080044GO:0047218,GO:0052696,GO:0016757,GO:0043231,GO:0009813,GO:0010224,GO:0010224
+		Ccrd_v2_14134_g10	Similar to CRK10 Cysteine-rich receptor-like protein kinase 10 (Arabidopsis thaliana)	AT4G23180.1	GO:0005524,GO:0016021,GO:0004674,GO:0005886,GO:0016301,GO:0042742,GO:0006468,GO:0009506
+		Ccrd_v2_14151_g10	Protein of unknown function	AT2G22010.1	GO:0016021,GO:0005737,GO:0060154,GO:0008270,GO:0051603,GO:0016567,GO:0034450,GO:0009615,GO:0051726,GO:0016874,GO:0016874,GO:0016567,GO:0006511,GO:0030163,GO:0000151,GO:0004842,GO:0005737,GO:0008270
+		Ccrd_v2_18020_g12	Protein of unknown function	AT1G60270.1	GO:0102483,GO:0005975,GO:1901657,GO:0005576,GO:0008422
+		Ccrd_v2_23056_g16	Similar to LARP6A La-related protein 6A (Arabidopsis thaliana)	AT3G30390.2	GO:0005886,GO:0016021,GO:0005774,GO:0005773,GO:0005886,GO:0006865,GO:0005774,GO:0016020,GO:0015171,GO:0005773
+		Ccrd_v2_25981_scaffold_1866	Similar to TOR1 Microtubule-associated protein TORTIFOLIA1 (Arabidopsis thaliana)	AT4G27060.1	GO:0009826,GO:0010005,GO:0009507,GO:0008017,GO:0007275,GO:0010031
+		Ccrd_v2_27027_scaffold_4688	Similar to UGT85A24 7-deoxyloganetin glucosyltransferase (Gardenia jasminoides)	AT1G22360.1	GO:0008152,GO:0016757,GO:0016758,GO:0052696,GO:0043231,GO:0080043,GO:0005634,GO:0015020,GO:0008194,GO:0009813,GO:0005634,GO:0080044
+		Ccrd_v2_27640_scaffold_7282	Similar to At1g77330 1-aminocyclopropane-1-carboxylate oxidase 5 (Arabidopsis thaliana)	AT1G77330.1	GO:0009693,GO:0055114,GO:0051213,GO:0009693,GO:0005737,GO:0009815,GO:0006952,GO:0031418,GO:0046872
+		Ccrd_v2_27787_scaffold_8275	Similar to At4g27290 G-type lectin S-receptor-like serine/threonine-protein kinase At4g27290 (Arabidopsis thaliana)	AT4G27290.1	GO:0048544,GO:0030246,GO:0005524,GO:0005886,GO:0004672
+		Ccrd_v2_27878_scaffold_8980	Similar to KOR Endoglucanase 25 (Arabidopsis thaliana)	AT5G49720.1	GO:0005886,GO:0005886,GO:0005886,GO:0009735,GO:0030244,GO:0005794,GO:0008810,GO:0005794,GO:0005768,GO:0030244,GO:0048367,GO:0009826,GO:0005769,GO:0071555,GO:0043622,GO:0004553,GO:0016021,GO:0042538,GO:0009504,GO:0030245,GO:0005802,GO:0005794
+	**SP**	Ccrd_v2_08636_g05	"Protein of unknown function" AED:0.07 eAED:0.07 QI:2|1|0.71|1|0.66|0.57|7|1|266	AT2G42080	GO:0006457,GO:0051510,GO:0009644,GO:0042538,GO:1903578,GO:0005739
+	**VS**	Ccrd_v2_28301_scaffold_13518	"Similar to WAK5 Wall-associated receptor kinase 5 (Arabidopsis thaliana)"	AT1G21230	GO:0030247,GO:0007166,GO:0006468,GO:0006468,GO:0004674,GO:0004674,GO:0016301,GO:0005886,GO:0016021,GO:0005576,GO:0005509,GO:0005524
+	**VT**	Ccrd_v2_28074_scaffold_10747	"Similar to DAD2 Probable strigolactone esterase DAD2 (Petunia hybrida)"	AT3G03990	GO:0005737,GO:0003824,GO:0010223,GO:1902348,GO:1901601,GO:0005737,GO:0016787,GO:0005634
+	**C3**	Ccrd_v2_07924_g04	Similar to CNX1 Calnexin homolog 1 (Arabidopsis thaliana)	AT5G61790	GO:0009505,GO:0016021,GO:0006457,GO:0005739,GO:0005886,GO:0005739,GO:0051082,GO:0005773,GO:0009507,GO:0005789,GO:0043231,GO:0005783,GO:0030246,GO:0009506,GO:0005509,GO:0016020,GO:0005774,GO:0005773,GO:0005783
+		Ccrd_v2_27266_scaffold_5544	Protein of unknown function	AT5G03420	GO:0009507,GO:0016310,GO:0016301
+		Ccrd_v2_28195_scaffold_11814	Protein of unknown function	AT4G15030	GO:0003674,GO:0005634,GO:0005634,GO:0008150
-	**A41**	Ccrd_v2_04456_g02	Similar to IQD14 Protein IQ-DOMAIN 14 (Arabidopsis thaliana)	AT2G43680.1	GO:0005634,GO:0005634,GO:0005516,GO:0005634,GO:0005886,GO:0005886
-		Ccrd_v2_06154_g03	Similar to FUT11 Glycoprotein 3-alpha-L-fucosyltransferase A (Arabidopsis thaliana)	AT3G19280.1	GO:0016757,GO:0036065,GO:0006487,GO:0017083,GO:0032580,GO:0016021,GO:0008417,GO:0008417,GO:0005794,GO:0000139,GO:0008417,GO:0071555,GO:0046920
-		Ccrd_v2_06179_g03	Protein of unknown function	AT5G15120.1	GO:0005634,GO:0046872,GO:0070483,GO:0005634,GO:0017172,GO:0005829,GO:0009061,GO:0009061,GO:0001666,GO:0018171
-		Ccrd_v2_07181_g04	Similar to PAB8 Polyadenylate-binding protein 8 (Arabidopsis thaliana)	AT1G49760.2	GO:0000166,GO:0005634,GO:0003743,GO:0005515,GO:0006417,GO:0005737,GO:0003723,GO:0046686,GO:0003723,GO:0005737,GO:0016032,GO:0005829
-		Ccrd_v2_09339_g05	Similar to RPL7D 60S ribosomal protein L7-4 (Arabidopsis thaliana)	AT2G44120.1	GO:0009507,GO:0009507,GO:0022626,GO:0016020,GO:0022626,GO:0009507,GO:0009507,GO:0005737,GO:0009506,GO:0005773,GO:0016020,GO:0005730,GO:0015934,GO:0022625,GO:0022626,GO:0005730,GO:0005829,GO:0005737,GO:0003735,GO:0005773,GO:0005829,GO:0022626,GO:0022626,GO:0006412
-		Ccrd_v2_15954_g11	Similar to NCS1 S-norcoclaurine synthase 1 (Papaver somniferum)	AT3G06920.1	GO:0008150,GO:0005575
-		Ccrd_v2_18062_g12	Similar to SCPL49 Serine carboxypeptidase-like 49 (Arabidopsis thaliana)	AT3G10410.1	GO:0005773,GO:0005576,GO:0004185,GO:0006508,GO:0005773,GO:0005576,GO:0004185,GO:0005829,GO:0051603,GO:0005773
-		Ccrd_v2_20247_g13	Similar to SAUR24 Auxin-responsive protein SAUR24 (Arabidopsis thaliana)	AT4G38840.1	GO:0009409,GO:0009733,GO:0005886,GO:0003674,GO:0009733,GO:0009507
-		Ccrd_v2_21176_g15	Similar to D4H Deacetoxyvindoline 4-hydroxylase (Catharanthus roseus)	AT2G30830.1	GO:0055114,GO:0005737,GO:0009815,GO:0046872

-		Ccrd_v2_24251_g17	Protein of unknown function	AT5G48050.1	GO:0003674,GO:0005575,GO:0008150
-		Ccrd_v2_24348_g17	Protein of unknown function	AT1G51690.2	GO:0005737,GO:0000159,GO:0008601,GO:0000166,GO:0005737,GO:0005737
-		Ccrd_v2_24594_g17	Similar to DYW9 Pentatricopeptide repeat-containing protein At4g30700 (Arabidopsis thaliana)	AT4G30700.1	GO:0080156, GO:0005739, GO:0000963,GO:0003674,GO:0005739,GO:0009507,GO:0008270,GO:0016554
-		Ccrd_v2_26552_scaffold_3173	Similar to Arginine decarboxylase (Pisum sativum)	AT4G34710.2	GO:0009753,GO:0006527,GO:0048316,GO:0005634,GO:0008295,GO:0009793,GO:0006970,GO:0006596,GO:0009446,GO:0080167,GO:0008792,GO:0009737,GO:0009651,GO:0009409,GO:0009611,GO:0006979,GO:0006596,GO:0005634,GO:0033388
-		Ccrd_v2_27366_scaffold_5971	Similar to AGL62 Agamous-like MADS-box protein AGL62 (Arabidopsis thaliana)	AT5G60440.1	GO:0003700,GO:0003700,GO:0003677,GO:0006351,GO:0046983,GO:0003677,GO:0006355,GO:0009960,GO:0005634,GO:0005634,GO:0003700
-		Ccrd_v2_27604_scaffold_7146	Protein of unknown function	AT5G11260.1	GO:0010224,GO:0005515,GO:0005515,GO:0005634,GO:0005515,GO:0006355,GO:0005515,GO:0003700,GO:0005634,GO:0005515,GO:0006351,GO:0042753,GO:0009737,GO:0005515,GO:0009740,GO:0005515,GO:0010114,GO:0031539,GO:0003700,GO:0009585,GO:0010224,GO:0006355,GO:0003690,GO:0003677,GO:0080167,GO:0005634,GO:0010017,GO:0006355,GO:0010224,GO:0009963,GO:0005634,GO:0003700,GO:0003677,GO:0005634,GO:0005634,GO:0005515,GO:0009640,GO:0043565,GO:0010218,GO:0003700,GO:0005515,GO:0009640,GO:0010099
-		Ccrd_v2_27653_scaffold_7339	Similar to ATPC2 ATP synthase gamma chain 2, chloroplastic (Arabidopsis thaliana)	AT1G15700.1	GO:0046933,GO:0009507,GO:0009507,GO:0045261,GO:2000067,GO:0015986,GO:0009507,GO:0009544,GO:0046961,GO:0030234,GO:0046933,GO:0009507
-		Ccrd_v2_27689_scaffold_7631	Similar to HSP70-3 Heat shock 70 kDa protein 3 (Arabidopsis thaliana)	AT3G09440.2	GO:0005794,GO:0046686,GO:0048046,GO:0005886,GO:0005773,GO:0009408,GO:0002020,GO:0009506,GO:0009615,GO:0005515,GO:0005829,GO:0009408,GO:0016363,GO:0005774,GO:0080167,GO:0005524,GO:0005794,GO:0022626,GO:0009507,GO:0006457,GO:0005618,GO:0005886,GO:0005829
-		Ccrd_v2_28031_scaffold_10306	Protein of unknown function	AT1G09910.1	GO:0005975,GO:0016829,GO:0005576,GO:0016829,GO:0030246
-		Ccrd_v2_28038_scaffold_10336	Similar to ABCC3 ABC transporter C family member 3 (Arabidopsis thaliana)	AT3G13080.1	GO:0042626,GO:0000325,GO:0005774,GO:0010290,GO:0005524,GO:0048046,GO:0005886,GO:0000325,GO:0005774,GO:0005886,GO:0055085,GO:0048046,GO:0015431,GO:0042626,GO:0008559,GO:0005774,GO:0000325,GO:0005524,GO:0048046,GO:0016021,GO:0009506,GO:0005886,GO:0005773,GO:0005774,GO:0016021,GO:0048046,GO:0042626,GO:0000325,GO:0005774,GO:0005886,GO:0004867,GO:0005576,GO:0002213,GO:0006952,GO:0006508,GO:0009611,GO:0009611,GO:0004867,GO:0008233,GO:0050832
-		Ccrd_v2_28135_scaffold_11330	Similar to Glu S.griseus protease inhibitor (Momordica charantia)	AT5G43580.1	GO:0004867,GO:0005576,GO:0002213,GO:0006952,GO:0006508,GO:0009611,GO:0009611,GO:0004867,GO:0008233,GO:0050832
-		Ccrd_v2_28245_scaffold_12402	Similar to NUP98B Nuclear pore complex protein NUP98B (Arabidopsis thaliana)	AT1G59660.1	GO:0051028,GO:0005215,GO:0005635,GO:0005634,GO:0005515
-	**SP**	Ccrd_v2_00612_g01	Similar to At3g12360 Ankyrin repeat-containing protein At3g12360 (Arabidopsis thaliana)	AT3G18670.1	GO:0016021
-		Ccrd_v2_02223_g01	Similar to ATPC2 ATP synthase gamma chain 2, chloroplastic (Arabidopsis thaliana)	AT1G15700.1	GO:0046933,GO:0009507,GO:0009507,GO:0045261,GO:2000067,GO:0015986,GO:0009507,GO:0009544,GO:0046961,GO:0030234,GO:0046933,GO:0009507
-		Ccrd_v2_05137_g02	Similar to CLC-F Chloride channel protein CLC-f (Arabidopsis thaliana)	AT1G55620.2	GO:0009507,GO:0006821,GO:0005247,GO:0005794,GO:0005794,GO:0005247,GO:1902476,GO:0016020,GO:0005794,GO:0005247,GO:0031404,GO:0034707
-		Ccrd_v2_07224_g04	Similar to ADF1 Actin-depolymerizing factor 1 (Petunia hybrida)	AT5G59890.1	,GO:0030042,GO:0005737,GO:0042742,GO:0015629,GO:0005622,GO:0005737,GO:0003779,GO:0005737,GO:0009870
-		Ccrd_v2_08493_g05	Protein of unknown function	AT3G47160.2	,GO:0005634,GO:0005634,GO:0008270
-		Ccrd_v2_08786_g05	Similar to Os04g0444900 PHD finger protein ALFIN-LIKE 4 (Oryza sativa subsp. japonica)	AT5G20510.1	GO:0016568,GO:0005634,GO:0005634,GO:0005634,GO:0006355,GO:0009414,GO:0006351,GO:0035064,GO:0042393,GO:0003677,GO:0006355,GO:0003714,GO:0003677,GO:0008270,GO:0009651
-		Ccrd_v2_09249_g05	Similar to FUT11 Glycoprotein 3-alpha-L-fucosyltransferase A (Arabidopsis thaliana)	AT3G19280.1	GO:0016757,GO:0036065,GO:0006487,GO:0017083,GO:0032580,GO:0016021,GO:0008417,GO:0008417,GO:0005794,GO:0000139,GO:0008417,GO:0071555,GO:0046920
-		Ccrd_v2_09299_g05	Protein of unknown function	AT4G24810.3	GO:0004672,GO:0005886,GO:0005524,GO:0009507,GO:0005886,GO:0006468,GO:0005886,GO:0009507
-		Ccrd_v2_10077_g06	Similar to At1g11050 Probable receptor-like protein kinase At1g11050 (Arabidopsis thaliana)	AT1G11050.1	GO:0005524,GO:0005886,GO:0016301,GO:0016301,GO:0006468,GO:0016021
-		Ccrd_v2_10412_g06	Similar to CLC-F Chloride channel protein CLC-f (Arabidopsis thaliana)	AT1G55620.2	GO:0009507,GO:0006821,GO:0005247,GO:0005794,GO:0005794,GO:0005247,GO:1902476,GO:0016020,GO:0005794,GO:0005247,GO:0031404,GO:0034707
-		Ccrd_v2_13136_g09	Protein of unknown function	AT5G52380.1	GO:0003676,GO:0008270,GO:0005634,GO:0003676
-		Ccrd_v2_13628_g09	Similar to At3g01520 Universal stress protein A-like protein (Arabidopsis thaliana)	AT2G47710.1	GO:0005794,GO:0005886,GO:0005773,GO:0016787,GO:0006950
-		Ccrd_v2_15785_g11	Protein of unknown function	AT1G51810.1	GO:0005886,GO:0016301
-		Ccrd_v2_19573_g13	Similar to CYP80B3 (S)-N-methylcoclaurine 3'-hydroxylase isozyme 1 (Fragment) (Papaver somniferum)	AT5G07990.1	GO:0016020,GO:0044550,GO:0009733,GO:0055114,GO:0005506,GO:0016711,GO:0009813,GO:0005576,GO:0020037,GO:0009411,GO:0016711,GO:0019825,GO:0016711,GO:0005789,GO:0009813,GO:0016021
-	**VS**	Ccrd_v2_03886_g02	Similar to CXE6 Probable carboxylesterase 6 (Arabidopsis thaliana)	AT5G16080.1	GO:0009056,GO:0016787,GO:0005575,GO:0008152,GO:0052689
-		Ccrd_v2_04176_g02	Similar to 3-isopropylmalate dehydrogenase, chloroplastic (Brassica napus)	AT5G14200.2	GO:0009651,GO:0005737,GO:0009651,GO:0051287,GO:0055114,GO:0009098,GO:0009536,GO:0003862,GO:0009570,GO:0003862,GO:0009570,GO:0055114,GO:0003862,GO:0019761,GO:0009651,GO:0016616,GO:0055114,GO:0009507,GO:0000287,GO:0009507,GO:0009570,GO:0009507,GO:0009098,GO:0009507,GO:0009507,GO:0009507
-		Ccrd_v2_06153_g03	Similar to CHSP70 Stromal 70 kDa heat shock-related protein, chloroplastic (Fragment) (Spinacia oleracea)	AT5G49910.1	GO:0005524,GO:0006457,GO:0009570,GO:0006457,GO:0009507,GO:0051082,GO:0009570,GO:0045036,GO:0005622,GO:0009532,GO:0005623,GO:0009579,GO:0046686,GO:0005515,GO:0009941,GO:0009408,GO:0009532,GO:0048046,GO:0009570,GO:0009507
-		Ccrd_v2_16451_g11	Similar to FBL23 Putative F-box/LRR-repeat protein 23 (Arabidopsis thaliana)	AT1G76620.1	GO:0003674,GO:0008150
-		Ccrd_v2_18490_g13	Protein of unknown function	AT1G24300.1	GO:0005737,GO:0009507,GO:0005737
-		Ccrd_v2_23230_g16	Similar to Os07g0607800 Acidic leucine-rich nuclear phosphoprotein 32-related protein 1 (Oryza sativa subsp. japonica)	AT3G50690.1	GO:0008150,GO:0005634,GO:0003674
-		Ccrd_v2_25081_scaffold_489	Protein of unknown function	AT5G62960.1	GO:0003674,GO:0016021,GO:0008150,GO:0009507
-		Ccrd_v2_25494_scaffold_1004	Similar to TTL1 TPR repeat-containing thioredoxin TTL1 (Arabidopsis thaliana)	AT1G53300.1	GO:0045454,GO:0009738,GO:0005634,GO:0006970,GO:0006970,GO:0009737,GO:0009651,GO:0009789
-		Ccrd_v2_26689_scaffold_3595	Protein of unknown function	AT5G62960.1	GO:0003674,GO:0016021,GO:0008150,GO:0009507

-		Ccrd_v2_26876_scaffold_4192	Protein of unknown function	AT5G25770.1	GO:0009507,GO:0016787,GO:0008150
-		Ccrd_v2_27108_scaffold_4955	Protein of unknown function	AT4G35540.1	GO:0003690,GO:0005634,GO:0009846,GO:0070063,GO:0006352,GO:0001093,GO:0006355,GO:0005634,GO:0009793,GO:0008270
-		Ccrd_v2_27600_scaffold_7131	Protein of unknown function	AT1G03060.1	GO:0000932,GO:0071472,GO:0009737,GO:0007033,GO:0005515,GO:0005634,GO:0010090,GO:0009825,GO:0033962,GO:1904580,GO:0007165
-		Ccrd_v2_27854_scaffold_8723	Protein of unknown function	AT2G07360.2	GO:0005886,GO:0009507,GO:0005634,GO:0009507,GO:0005829,GO:0016020,GO:0005515
-		Ccrd_v2_28062_scaffold_10541	Similar to At1g67340 F-box protein At1g67340 (Arabidopsis thaliana)	AT1G67340.1	GO:0005634,GO:0016567
-					
	**VT**	Ccrd_v2_01706_g01	Protein of unknown function	AT5G64480.1	GO:0003674,GO:0008150
-		Ccrd_v2_02539_g01	Similar to APK1B Protein kinase APK1B, chloroplastic (Arabidopsis thaliana)	AT2G17220.1	GO:0009507,GO:0006468,GO:0004674,GO:0016301,GO:0005886,GO:0009507,GO:0005524
-		Ccrd_v2_02540_g01	Similar to POPTRDRAFT_820933 CASP-like protein 1D1 (Populus trichocarpa)	AT4G15610.1	GO:0005886,GO:0005794,GO:0005768,GO:0005794,GO:0005886,GO:0005802,GO:0016021,GO:0005794
-		Ccrd_v2_04023_g02	Protein of unknown function	AT1G71820.2	GO:0005829,GO:0005515,GO:0005829,GO:0006887,GO:0000145,GO:0009524,GO:0005576,GO:0005737,GO:0000149,GO:0005856,GO:0009506,GO:0009846,GO:0006887,GO:0005886,GO:0051601,GO:0009860,GO:0000145,GO:0005618,GO:0005886,GO:0005737,GO:0060321
-		Ccrd_v2_05571_g03	Similar to Triosephosphate isomerase, cytosolic (Fragment) (Lactuca sativa)	AT3G55440.1	GO:0006096,GO:0005886,GO:0005737,GO:0005739,GO:0009651,GO:0005739,GO:0005829,GO:0005886,GO:0009735,GO:0009570,GO:0009507,GO:0006094,GO:0009506,GO:0005829,GO:0046166,GO:0006098,GO:0046686,GO:0005618,GO:0010043,GO:0006094,GO:0004807,GO:0009570,GO:0005507,GO:0005774,GO:0006096,GO:0048046,GO:0004807,GO:0006096,GO:0044262,GO:0018119,GO:0005773,GO:0019563
-		Ccrd_v2_06726_g03	Similar to GXM2 Glucuronoxylan 4-O-methyltransferase 2 (Arabidopsis thaliana)	AT1G71690.1	GO:0045492,GO:0016021,GO:0008150,GO:0003674,GO:0005576
-		Ccrd_v2_08395_g05	Similar to NUDT7 Nudix hydrolase 7 (Arabidopsis thaliana)	AT4G12720.4	GO:0047631,GO:0005829,GO:0005515,GO:0005737,GO:0005634,GO:0010581,GO:0009870,GO:0046872,GO:0047631,GO:0000210,GO:0047631,GO:0042742,GO:0005829,GO:0051287,GO:0009651,GO:0080041,GO:0005634,GO:0017110,GO:0005634,GO:0000210,GO:0006979,GO:0010193,GO:0005829,GO:0005829,GO:0005634,GO:0016787,GO:0009626,GO:0070212,GO:0080041,GO:0005886,GO:0016787,GO:0005634,GO:0080042
-		Ccrd_v2_08691_g05	Similar to BHLH3 Transcription factor bHLH3 (Arabidopsis thaliana)	AT4G16430.1	GO:0006355,GO:0005634,GO:0046983,GO:0003677,GO:0003700,GO:0003677,GO:0005634,GO:0006351,GO:0003700
-		Ccrd_v2_12280_g08	Similar to WAK2 Wall-associated receptor kinase 2 (Arabidopsis thaliana)	AT1G21270.1	GO:0005886,GO:0005623,GO:0004674,GO:0009992,GO:0030247,GO:0016021,GO:0007166,GO:0006468,GO:0009992,GO:0006468,GO:0005524,GO:0009826,GO:0009751,GO:0005509,GO:0004674,GO:0009311
-		Ccrd_v2_12994_g09	Similar to PAO5 Probable polyamine oxidase 5 (Arabidopsis thaliana)	AT1G65840.1	GO:0055114,GO:0052895,GO:0052894,GO:0046592,GO:0008131,GO:0052901,GO:0006598,GO:0005777,GO:0005634,GO:0046592,GO:0006598
-		Ccrd_v2_14244_g10	Similar to atpB ATP synthase subunit beta, chloroplastic (Ostreococcus tauri)	ATCG00480.1	GO:0009535,GO:0046933,GO:0005618,GO:0009535,GO:0009544,GO:0031977,GO:0009534,GO:0009570,GO:0010287,GO:0015991,GO:0016020,GO:0015986,GO:0005524,GO:0009507,GO:0008270,GO:0010287,GO:0009570,GO:0045261,GO:0015986,GO:0009817,GO:0009535,GO:0009507,GO:0005754,GO:0009579,GO:0009579,GO:0010319,GO:0009409,GO:0009579,GO:0015078,GO:0009579,GO:0009535
-		Ccrd_v2_15486_g10	Similar to PCMP-H82 Pentatricopeptide repeat-containing protein At3g62890 (Arabidopsis thaliana)	AT4G33170.1	GO:0008270,GO:0005739
-		Ccrd_v2_15956_g11	Similar to DGK7 Diacylglycerol kinase 7 (Arabidopsis thaliana)	AT4G30340.1	GO:0016310,GO:0005886,GO:0004143,GO:0048366,GO:0048364,GO:0005524,GO:0007205,GO:0006952,GO:0005634,GO:0007205,GO:0004143
-		Ccrd_v2_16785_g11	Protein of unknown function	AT5G02370.1	GO:0009507,GO:0005874,GO:0008017,GO:0005794,GO:0005524,GO:0003777
-		Ccrd_v2_18295_g12	Protein of unknown function	AT2G39360.1	GO:0005524,GO:0016301,GO:0005886,GO:0005886,GO:0016021,GO:0004674,GO:0006468,GO:0005886,GO:0016301
-		Ccrd_v2_19314_g13	Similar to EXPA15 Expansin-A15 (Arabidopsis thaliana)	AT2G03090.1	GO:0009664,GO:0016020,GO:0005576,GO:0009831,GO:0009826,GO:0005618,GO:0009828,GO:0009826,GO:0006949,GO:0005576,GO:0009828
-		Ccrd_v2_24102_g17	Similar to IQM2 IQ domain-containing protein IQM2 (Arabidopsis thaliana)	AT3G13600.1	GO:0005516,GO:0008150,GO:0005737,GO:0005634,GO:0005634
-		Ccrd_v2_25133_scaffold_555	Similar to FUT11 Glycoprotein 3-alpha-L-fucosyltransferase A (Arabidopsis thaliana)	AT3G19280.1	GO:0016757,GO:0036065,GO:0006487,GO:0017083,GO:0032580,GO:0016021,GO:0008417,GO:0008417,GO:0005794,GO:0000139,GO:0008417,GO:0071555,GO:0046920
-		Ccrd_v2_26093_scaffold_2092	Similar to CYP71A6 Cytochrome P450 71A6 (Fragment) (Nepeta racemosa)	AT3G26210.1	GO:0016021,GO:0005506,GO:0044550,GO:0019825,GO:0055114,GO:0098542,GO:0016709,GO:0016020,GO:0020037
-		Ccrd_v2_26094_scaffold_2092	Similar to CYP71A4 Cytochrome P450 71A4 (Solanum melongena)	AT3G48290.2	GO:0019825,GO:0055114,GO:0016709,GO:0005506,GO:0020037,GO:0016021,GO:0004497,GO:0055114,GO:0044550,GO:0016020,GO:0016705
-		Ccrd_v2_26586_scaffold_3252	Similar to PCMP-E73 Pentatricopeptide repeat-containing protein At2g36980, mitochondrial (Arabidopsis thaliana)	AT2G36980.1	GO:0005739,GO:0005739,GO:0008150
-		Ccrd_v2_26677_scaffold_3537	Similar to GTE10 Transcription factor GTE10 (Arabidopsis thaliana)	AT5G63320.3	GO:0005634,GO:0005634,GO:0009738,GO:0009651,GO:0005515,GO:0005634,GO:0009737,GO:0051365,GO:0005515,GO:0006351,GO:0045892,GO:0009409,GO:0005634
-		Ccrd_v2_26890_scaffold_4225	Similar to RPS14 40S ribosomal protein S14 (Fragment) (Nicotiana tabacum)	AT3G11510.1	GO:0005730,GO:0003735,GO:0022627,GO:0022626,GO:0006412,GO:0022627,GO:0005737,GO:0022626,GO:0022626
-		Ccrd_v2_27588_scaffold_7107	Similar to At4g20940 Probable LRR receptor-like serine/threonine-protein kinase At4g20940 (Arabidopsis thaliana)	AT1G74190.1	GO:0016021,GO:0007165
-		Ccrd_v2_27798_scaffold_8336	Protein of unknown function	AT4G32420.3	GO:0005634,GO:0005634,GO:0005634,GO:0006457,GO:0003755
-		Ccrd_v2_28217_scaffold_12100	Similar to EIF5 Eukaryotic translation initiation factor 5 (Phaseolus vulgaris)	AT1G36730.1	GO:0006446,GO:0003743,GO:0005829,GO:0005525,GO:0003743,GO:0006413
-	**C3**	Ccrd_v2_04039_g02	Protein of unknown function	AT2G05642.1	GO:0009507,GO:0003674,GO:0008150
-		Ccrd_v2_26193_scaffold_2351	Similar to Horcolin (Hordeum vulgare var. distichum)	AT1G19715.1	GO:0005576,GO:0005576,GO:0030246,GO:0005576
-		Ccrd_v2_27064_scaffold_4803	Similar to XTH9 Xyloglucan endotransglucosylase/hydrolase protein 9 (Arabidopsis thaliana)	AT4G03210.1	GO:0016762,GO:0010411,GO:0005618,GO:0016798,GO:0048046,GO:0005618,GO:0016762,GO:0071555,GO:0042546,GO:0005576,GO:0071555,GO:0048046,GO:0004553
-		Ccrd_v2_27844_scaffold_8667	Protein of unknown function	AT1G49360.1	GO:0005634,GO:0008150,GO:0003674

Supplementary Table S5 | GO-term enrichment in PAV associated regions. Only PAV specifically present in one genotype and absent in the other were considered.

GO terms	GO Category	REFLIST (26684)	A41 presence PAV (14 genes)	expected	fold Enrichment	p-value
cellular process regulating host cell cycle in response to virus (GO:0060154)	P	1	1	.00	> 100	5.25E-04
circumnutation (GO:0010031)	P	4	1	.00	> 100	2.10E-03
multicellular organismal movement (GO:0050879)	P	4	1	.00	> 100	2.10E-03
cellular response to virus (GO:0098586)	P	13	1	.01	> 100	6.80E-03
signal peptide processing (GO:0006465)	P	17	1	.01	> 100	8.88E-03
flavonoid glucuronidation (GO:0052696)	P	58	2	.03	65.72	4.23E-04
cellular glucuronidation (GO:0052695)	P	58	2	.03	65.72	4.23E-04
glucuronate metabolic process (GO:0019585)	P	58	2	.03	65.72	4.23E-04
uronic acid metabolic process (GO:0006063)	P	60	2	.03	63.53	4.52E-04
protein processing (GO:0016485)	P	38	1	.02	50.16	1.98E-02
recognition of pollen (GO:0048544)	P	39	1	.02	48.87	2.03E-02
cell recognition (GO:0008037)	P	39	1	.02	48.87	2.03E-02
protein maturation (GO:0051604)	P	43	1	.02	44.33	2.23E-02
pollen-pistil interaction (GO:0009875)	P	44	1	.02	43.32	2.28E-02
response to UV-B (GO:0010224)	P	47	1	.02	40.55	2.44E-02
flavonoid biosynthetic process (GO:0009813)	P	101	2	.05	37.74	1.26E-03
response to virus (GO:0009615)	P	53	1	.03	35.96	2.75E-02
flavonoid metabolic process (GO:0009812)	P	107	2	.06	35.63	1.42E-03
amino acid transport (GO:0006865)	P	57	1	.03	33.44	2.95E-02
monosaccharide metabolic process (GO:0005996)	P	133	2	.07	28.66	2.17E-03
response to light intensity (GO:0009642)	P	76	1	.04	25.08	3.91E-02
response to UV (GO:0009411)	P	78	1	.04	24.44	4.02E-02
response to toxic substance (GO:0009636)	P	86	1	.05	22.16	4.42E-02
organic acid transport (GO:0015849)	P	89	1	.05	21.42	4.57E-02
carboxylic acid transport (GO:0046942)	P	89	1	.05	21.42	4.57E-02
regulation of cell cycle (GO:0051726)	P	90	1	.05	21.18	4.62E-02
monocarboxylic acid metabolic process (GO:0032787)	P	392	2	.21	9.72	1.75E-02
single-organism carbohydrate metabolic process (GO:0044723)	P	409	2	.21	9.32	1.89E-02
response to light stimulus (GO:0009416)	P	422	2	.22	9.03	2.01E-02
response to radiation (GO:0009314)	P	442	2	.23	8.62	2.19E-02
carbohydrate metabolic process (GO:0005975)	P	829	3	.43	6.90	8.44E-03
multi-organism process (GO:0051704)	P	976	3	.51	5.86	1.32E-02
proteolysis (GO:0006508)	P	662	2	.35	5.76	4.60E-02
protein modification process (GO:0036211)	P	1599	3	.84	3.58	4.76E-02
cellular protein modification process (GO:0006464)	P	1599	3	.84	3.58	4.76E-02
cellular protein metabolic process (GO:0044267)	P	2256	4	1.18	3.38	2.57E-02
protein metabolic process (GO:0019538)	P	2606	4	1.37	2.93	4.09E-02
primary metabolic process (GO:0044238)	P	6218	7	3.26	2.15	2.66E-02
organic substance metabolic process (GO:0071704)	P	6636	7	3.48	2.01	3.73E-02
ubiquitin-ubiquitin ligase activity (GO:0034450)	F	2	1	.00	> 100	1.05E-03
sinapate 1-glucosyltransferase activity (GO:0050284)	F	4	1	.00	> 100	2.10E-03
hydroxycinnamate 4-beta-glucosyltransferase activity (GO:0047218)	F	5	1	.00	> 100	2.62E-03

glucuronosyltransferase activity (GO:0015020)	F	9	1	.00	> 100	4.71E-03
quercetin 7-O-glucosyltransferase activity (GO:0080044)	F	57	2	.03	66.88	4.08E-04
quercetin 3-O-glucosyltransferase activity (GO:0080043)	F	57	2	.03	66.88	4.08E-04
UDP-glucosyltransferase activity (GO:0035251)	F	99	2	.05	38.51	1.22E-03
protein heterodimerization activity (GO:0046982)	F	55	1	.03	34.65	2.85E-02
amino acid transmembrane transporter activity (GO:0015171)	F	55	1	.03	34.65	2.85E-02
beta-glucosidase activity (GO:0008422)	F	56	1	.03	34.04	2.90E-02
glucosyltransferase activity (GO:0046527)	F	113	2	.06	33.73	1.58E-03
glucosidase activity (GO:0015926)	F	63	1	.03	30.25	3.26E-02
ubiquitin protein ligase activity (GO:0061630)	F	72	1	.04	26.47	3.71E-02
ubiquitin-like protein ligase activity (GO:0061659)	F	73	1	.04	26.11	3.76E-02
carboxylic acid transmembrane transporter activity (GO:0046943)	F	74	1	.04	25.76	3.81E-02
organic acid transmembrane transporter activity (GO:0005342)	F	74	1	.04	25.76	3.81E-02
serine-type endopeptidase activity (GO:0004252)	F	78	1	.04	24.44	4.02E-02
microtubule binding (GO:0008017)	F	81	1	.04	23.53	4.17E-02
tubulin binding (GO:0015631)	F	87	1	.05	21.91	4.47E-02
UDP-glycosyltransferase activity (GO:0008194)	F	204	2	.11	18.69	5.00E-03
transferase activity, transferring hexosyl groups (GO:0016758)	F	298	2	.16	12.79	1.04E-02
transferase activity, transferring glycosyl groups (GO:0016757)	F	492	2	.26	7.75	2.67E-02
transferase activity (GO:0016740)	F	2876	5	1.51	3.31	1.26E-02
cortical microtubule, transverse to long axis (GO:0010005)	C	5	1	.00	> 100	2.62E-03
cortical microtubule (GO:0055028)	C	14	1	.01	> 100	7.32E-03
cytoplasmic microtubule (GO:0005881)	C	15	1	.01	> 100	7.84E-03
cortical microtubule cytoskeleton (GO:0030981)	C	17	1	.01	> 100	8.88E-03
cortical cytoskeleton (GO:0030863)	C	18	1	.01	> 100	9.40E-03
nucleosome (GO:0000786)	C	28	1	.01	68.07	1.46E-02
DNA packaging complex (GO:0044815)	C	31	1	.02	61.48	1.61E-02
cell cortex part (GO:0044448)	C	45	1	.02	42.36	2.34E-02
protein-DNA complex (GO:0032993)	C	47	1	.02	40.55	2.44E-02
cell cortex (GO:0005938)	C	51	1	.03	37.37	2.64E-02
cytoplasmic region (GO:0099568)	C	51	1	.03	37.37	2.64E-02
chromatin (GO:0000785)	C	72	1	.04	26.47	3.71E-02
cell periphery (GO:0071944)	C	3182	5	1.67	2.99	1.91E-02
plasma membrane (GO:0005886)	C	2725	4	1.43	2.80	4.72E-02

Supplementary Table S6 | Total number of miRNA loci identified using Mirena[7] for each of the 6 genotypes, together with the number of non-redundant miRNAs and pre-miRNAs.

	miRNA loci	unique miRNA	unique pre-miRNA	miRNA families
**2C**	241	143	211	45
**A41**	188	115	163	40
**VS**	74	51	67	22
**VT**	113	76	100	34
**C3**	219	133	192	45
**SP**	126	81	108	32

Supplementary Table S7 | Total number of miRNA:RNA interactions **(**Tapir[8]**)**, together with the number of miRNA families, non-redundant miRNAs and target transcripts involved, for each of the 6 genotypes.

**Genotype**	**miRNA:mRNA interactions**	**miRNA families**	**Unique miRNAs**	**Unique targets (% of genes with IPR)**
**2C**	1167	45	124	925 (89%)
**A41**	730	39	96	575 (91.4%)
**VS**	307	20	44	261 (91%)
**VT**	571	30	62	474 (91%)
**C3**	1087	45	116	862 (89.8%)
**SP**	322	28	59	241 (91%)

Supplementary Table S8 | Total target genes for identified 6 genotypes miRNA families using Tapir[8].

**Reference**		**A41**		**VS**		**VT**		**C3**		**SP**	
**miRNA family**	**count**	**miRNA family**	**count**	**miRNA family**	**count**	**miRNA family**	**count**	**miRNA family**	**count**	**miRNA family**	**count**
miR172	282	miR172	194	miR172	83	miR172	188	miR172	283	miR156	74
miR156	103	miR156	87	miR166	42	miR2630	68	miR156	103	miR166	29
miR2673	86	miR166	85	miR164	30	miR396	43	miR2673	86	miR7699	26
miR2630	73	miR1446	36	miR156	23	miR169	40	miR2630	73	miR9470	25
miR166	68	miR399	27	miR7699	18	miR166	30	miR166	69	miR171	17
miR396	53	miR171	26	miR157	16	miR7699	26	miR396	52	miR169	16
miR444	48	miR7699	23	miR169	16	miR2628	19	miR2079	37	miR5751	16
miR7699	41	miR1030	22	miR399	14	miR5247	17	miR1446	35	miR394	12
miR2079	38	miR396	22	miR396	13	miR3633	15	miR7699	35	miR396	12
miR1446	35	miR529	20	miR394	10	miR319	14	miR164	31	miR2628	11
miR164	31	miR393	18	miR171	9	miR156	11	miR169	27	miR393	10
miR171	28	miR444	17	miR167	8	miR171	11	miR393	25	miR319	9
miR169	27	miR169	15	miR530	8	miR393	11	miR1030	22	miR399	9
miR393	27	miR319	15	miR168	4	miR394	9	miR171	19	miR167	8
miR1030	26	miR397	13	miR902	4	miR1155	8	miR157	18	miR530	8
miR399	22	miR394	11	miR2680	3	miR530	7	miR399	18	miR1030	7
miR157	18	miR5258	10	miR398	2	miR399	6	miR5751	17	miR160	6
miR5751	17	miR167	9	miR7696	2	miR403	6	miR319	16	miR5254	6
miR319	16	miR168	9	miR1155	1	miR5254	6	miR397	13	miR390	5
miR397	13	miR395	8	miR160	1	miR1030	5	miR160	12	miR168	4
miR160	12	miR160	7	-	-	miR160	5	miR394	12	miR2658	4
miR394	11	miR2657	7	-	-	miR2651	5	miR167	10	miR2651	2
miR5258	11	miR530	7	-	-	miR390	5	miR2657	7	miR1155	1
miR167	10	miR164	5	-	-	miR408	4	miR530	7	miR159	1
miR168	8	miR408	4	-	-	miR824	4	miR902	7	miR403	1
miR2657	7	miR7696	4	-	-	miR164	3	miR5254	6	miR479	1
miR530	7	miR2630	3	-	-	miR5258	2	miR2680	5	miR7696	1
miR5254	6	miR2658	3	-	-	miR159	1	miR824	5	miR837	1
miR2680	5	miR2680	3	-	-	miR479	1	miR168	4	-	-
miR824	5	miR390	3	-	-	miR7696	1	miR408	4	-	-
miR408	4	miR902	3	-	-	-	-	miR2658	3	-	-
miR902	4	miR1155	2	-	-	-	-	miR390	3	-	-
miR2658	3	miR2651	2	-	-	-	-	miR5745	3	-	-
miR390	3	miR398	2	-	-	-	-	miR1155	2	-	-
miR1155	2	miR4414	2	-	-	-	-	miR2651	2	-	-
miR2651	2	miR479	2	-	-	-	-	miR398	2	-	-
miR398	2	miR5559	2	-	-	-	-	miR479	2	-	-
miR4414	2	miR3633	1	-	-	-	-	miR5559	2	-	-
miR479	2	miR403	1	-	-	-	-	miR6024	2	-	-
miR5559	2	-	-	-	-	-	-	miR7696	2	-	-
miR7696	2	-	-	-	-	-	-	miR837	2	-	-
miR837	2	-	-	-	-	-	-	miR159	1	-	-
miR159	1	-	-	-	-	-	-	miR3633	1	-	-
miR3633	1	-	-	-	-	-	-	miR403	1	-	-
miR403	1	-	-	-	-	-	-	miR4414	1	-	-

Supplementary Table S9 | SEACOMPARE from AGRIGO[3] of the GO terms enriched in the six genotypes for miRNA targets genes.

	GO Information			CM					2C		A41		VS		C3		SP	
No	GOTerm	Onto	Description	2C	A41	VS	C3	SP	FDR	Num	FDR	Num	FDR	Num	FDR	Num	FDR	Num
1	GO:0016070	P	RNA metabolic process						0.017	95	0.025	63	0.028	33	0.025	90	---	---
2	GO:0080090	P	regulation of primary metabolic process						0.03	65	0.033	46	0.0065	29	0.025	63	---	---
3	GO:0019222	P	regulation of metabolic process						0.03	67	0.033	47	0.0065	30	0.025	65	---	---
4	GO:0009698	P	phenylpropanoid metabolic process						0.03	6	0.002	6	---	---	0.025	6	---	---
5	GO:0060255	P	regulation of macromolecule metabolic process						0.03	66	0.033	46	0.0065	29	0.025	64	---	---
6	GO:0019439	P	aromatic compound catabolic process						0.03	7	0.0047	6	---	---	0.025	7	---	---
7	GO:0009889	P	regulation of biosynthetic process						0.03	63	0.033	44	0.0065	27	0.025	61	---	---
8	GO:0006355	P	regulation of transcription, DNA-dependent						0.03	63	0.033	44	0.0065	27	0.025	61	---	---
9	GO:0006350	P	transcription						0.03	72	0.033	50	0.0065	30	0.03	68	---	---
10	GO:0006351	P	transcription, DNA-dependent						0.03	72	0.033	50	0.0065	30	0.03	68	---	---
11	GO:0032774	P	RNA biosynthetic process						0.03	72	0.033	50	0.0065	30	0.03	68	---	---
12	GO:0009808	P	lignin metabolic process						0.03	6	0.0013	6	---	---	0.025	6	---	---
13	GO:0046274	P	lignin catabolic process						0.03	6	0.0013	6	---	---	0.025	6	---	---
14	GO:0046271	P	phenylpropanoid catabolic process						0.03	6	0.0013	6	---	---	0.025	6	---	---
15	GO:0051252	P	regulation of RNA metabolic process						0.03	63	0.033	44	0.0065	27	0.025	61	---	---
16	GO:0031326	P	regulation of cellular biosynthetic process						0.03	63	0.033	44	0.0065	27	0.025	61	---	---
17	GO:0045449	P	regulation of transcription						0.03	63	0.033	44	0.0065	27	0.025	61	---	---
18	GO:0042219	P	cellular amino acid derivative catabolic process						0.03	6	0.002	6	---	---	0.025	6	---	---
19	GO:0010467	P	gene expression						0.03	112	---	---	---	---	0.025	107	---	---
20	GO:0010556	P	regulation of macromolecule biosynthetic process						0.03	63	0.033	44	0.0065	27	0.025	61	---	---
21	GO:0010468	P	regulation of gene expression						0.03	64	0.043	44	0.0083	27	0.025	62	---	---
22	GO:0019219	P	regulation of nucleobase, nucleoside, nucleotide and nucleic acid metabolic process						0.03	63	0.033	44	0.0065	27	0.025	61	---	---
23	GO:0051171	P	regulation of nitrogen compound metabolic process						0.03	63	0.033	44	0.0065	27	0.025	61	---	---
24	GO:0006139	P	nucleobase, nucleoside, nucleotide and nucleic acid metabolic process						0.041	107	---	---	---	---	---	---	---	---
25	GO:0031323	P	regulation of cellular metabolic process						0.047	64	0.042	45	0.0065	28	0.037	62	---	---
26	GO:0007275	P	multicellular organismal development						0.047	10	---	---	0.0065	7	---	---	---	---
27	GO:0003676	F	nucleic acid binding						0.0034	153	0.034	100	0.0049	58	0.011	145	---	---
28	GO:0043227	C	membrane-bounded organelle						0.019	72	0.012	50	---	---	0.035	67	0.049	25
29	GO:0043231	C	intracellular membrane-bounded organelle						0.019	72	0.012	50	---	---	0.035	67	0.049	25
30	GO:0005634	C	nucleus						0.019	53	0.012	36	0.019	22	0.026	49	0.0018	23
31	GO:0019748	P	secondary metabolic process						---	---	0.033	7	---	---	---	---	---	---
32	GO:0006725	P	cellular aromatic compound metabolic process						---	---	0.048	10	---	---	---	---	---	---
33	GO:0005507	F	copper ion binding						---	---	0.034	9	---	---	---	---	---	---
34	GO:0005576	C	extracellular region						---	---	0.002	10	---	---	0.026	11	---	---
35	GO:0048046	C	apoplast						---	---	0.0028	7	---	---	---	---	---	---
36	GO:0032502	P	developmental process						---	---	---	---	0.021	7	---	---	---	---
37	GO:0044249	P	cellular biosynthetic process						---	---	---	---	0.034	49	---	---	---	---
38	GO:0003677	F	DNA binding						---	---	---	---	0.0027	42	---	---	---	---
39	GO:0009719	P	response to endogenous stimulus						---	---	---	---	---	---	---	---	0.045	7
40	GO:0010033	P	response to organic substance						---	---	---	---	---	---	---	---	0.045	7
41	GO:0009725	P	response to hormone stimulus						---	---	---	---	---	---	---	---	0.045	7

Supplementary Table S10 | Position of RGAs clusters identified for three of the main classes: RLK, RLP, NB and other-KTM.

Class	Region	2C	A41	VS	VT	C3	SP
**RLP**	CH1: 0-4Mb	15	15	12	11	14	12
	CH11: 1-3 Mb	9	10	9	9	9	7
**NB**	CH10: 16-18 Mb	-	-	4	-	-	5
	CH13: 40-41 Mb	5	5	4	3	5	4
**other-KTM**	CH10: 10-11 Mb	9	9	9	10	9	9

Supplementary Table S11 | SNP/INDEL statistics of reference and resequenced genotypes.

Genotype	Type	Total	Homozygous	Heterozygous
**2C (ref)**	SNP/INDEL	815,853	-	815,853
	SNP	781,53	-	781,53
	INDEL	34,323	-	34,323
	SNP/Indel rate	0.11%	-	0.11%
	SNP/INDELl/1000 bp	1.12	-	1.12
	1 SNP/INDEL every (bp)	892.85	-	892.85
**A41**	SNP/INDEL	6,344,545	1,816,814	4,527,731
	SNP	5,900,934	1,613,313	4,287,621
	INDEL	443,603	203,493	240,11
	SNP/Indel rate	0.87%	0.25%	0.62%
	SNP/INDELl/1000 bp	8.75	2.50	6.25
	1 SNP/INDEL every (bp)	114.27	399.05	160.12
**SP**	SNP/INDEL	14,391,656	6,219,192	8,172,464
	SNP	13,241,315	5,520,551	7,720,764
	INDEL	1,150,341	698,641	451,7
	SNP/Indel rate	1.98%	0.86%	1.12%
	SNP/INDELl/1000 bp	19.85	8.58	11.27
	1 SNP/INDEL every (bp)	50.37	116.57	88.71
**VS**	SNP/INDEL	14,495,680	5,235,727	9,259,953
	SNP	13,440,135	4,676,063	8,764,072
	INDEL	1,055,545	559,664	495,876
	SNP/Indel rate	1.99%	0.72%	1.27%
	SNP/INDELl/1000 bp	19.99	7.22	12.77
	1 SNP/INDEL every (bp)	50.01	138.47	78.29
**C3**	SNP/INDEL	12,847,630	4,415,152	8,432,478
	SNP	11,937,400	3,941,153	7,996,247
	INDEL	910,23	473,999	436,231
	SNP/Indel rate	1.77%	0.61%	1.16%
	SNP/INDELl/1000 bp	17.72	6.09	11.63
	1 SNP/INDEL every (bp)	56.43	164.20	85.97
**VT**	SNP/INDEL	12,848,307	5,470,477	7,377,830
	SNP	11,860,358	4,876,522	6,983,806
	INDEL	987,949	593,925	394,024
	SNP/Indel rate	1.77%	0.75%	1.02%
	SNP/INDELl/1000 bp	17.72	7.54	10.18
	1 SNP/INDEL every (bp)	56.42	132.52	98.26

Supplementary Table S12 | **Annotation of SNPs.** Number of homozygous and heterozygous effects by type and region.

Genotype	allelic state	Intergenic	Intronic	CDS	UTR	Downstream	Upstream	Others
A41	homoz.	1,470,424	289,569	47,438	14,697	445,064	453,978	4,293
	heteroz.	3,865,692	561,312	96,342	24,693	810,745	836,673	7,751
SP	homoz.	5,290,997	794,086	113,099	36,635	1,341,107	1,457,154	10,487
	heteroz.	7,254,162	771,315	127,513	33,373	1,210,759	1,287,122	10,544
VS	homoz.	4,458,599	662,258	96,437	30,683	1,090,508	1,162,823	8,96
	heteroz.	8,124,789	954,764	156,332	42,844	1,472,332	1,541,072	13,021
C3	homoz.	3,719,864	588,163	89,177	28,318	918,453	965,694	8,316
	heteroz.	7,299,755	946,618	161,191	44,089	1,443,850	1,488,796	13,304
VT	homoz.	4,602,895	736,982	108,712	35,177	1,231,373	1,320,127	1,158
	heteroz.	6,427,951	797,939	132,604	35,214	1,227,033	1,289,048	10,771

Supplementary Table S13 | Annotation of SNPs. Number of homozygous and heterozygous effects by functional class

Genotype		Missense	Nonsense	Silent
**A41**	homoz.	20,996 (45.5%)	244 (0.52%)	24,898 (53.9%)
	heteroz.	46,609 (49.4%)	943 (1%)	46,790 (49.6%)
**SP**	homoz.	51,163 (46.3%)	719 (0.65%)	58,514 (53.0%)
	heteroz.	62,675 (50.3%)	1,558 (1.3%)	60,352 (48.4%)
**VS**	homoz.	43,413 (46.1%)	553 (0.59%)	50,074 (53.2%)
	heteroz.	75,658 (49.5%)	1,775 (1.1%)	75,566 (49.4%)
**C3**	homoz.	39,827 (45.9%)	462 (0.53%)	46,396 (53.5%)
	heteroz.	77,217 (48.9%)	1,072 (1.1%)	78,922 (50.0%)
**VT**	homoz.	49,044 (46.2%)	669 (0.63%)	56,336 (53.1%)
	heteroz.	64,438 (49.6%)	1,534 (1.2%)	63,341 (49.2%)

Supplementary Table S14 | Variants with deleterious effect impact on protein function in resequenced genomes (*= no start codon). †V1_Genbank: nomenclature used in Scaglione et al[9]. ¥V2: nomenclature used in this paper.

†V1_Genbank	V2 nomeclature ¥	GENE CLASS	GENE	CHR	POS	REF	ALT	VARIANTS	Provean IMPACT	SCORE VALUE	A41	SP	VS	C3	VT	Reference
Ccrd_010165	Ccrd_v2_03867_g02	CQA	CH4	2	18883169	CA	CAAA	*4716 insAA	High	-9,275	ref	hetero	ref	ref	ref	ref
Ccrd_005870	Ccrd_v2_11622_g07	SL	GAS	7	11684843	C	A	L132F	High	-3,524	ref	hetero	hetero	ref	ref	ref
Ccrd_005870	Ccrd_v2_11622_g07	SL	GAS	7	11684357	A	G	Y168H	High	-4,905	ref	ref	hetero	hetero	hetero	ref
Ccrd_005870	Ccrd_v2_11622_g07	SL	GAS	7	11684323	A	T	Leu179*	High	-8,589	ref	ref	hetero	hetero	hetero	ref
Ccrd_005870	Ccrd_v2_11622_g07	SL	GAS	7	11684065	T	C	Q265R	High	-3,789	ref	hetero	hetero	hetero	hetero	ref
Ccrd_005870	Ccrd_v2_11622_g07	SL	GAS	7	11684045	G	C	L272V	High	-2,888	ref	hetero	ref	ref	ref	ref
Ccrd_005870	Ccrd_v2_11622_g07	SL	GAS	7	11683507	A	G	Y362H	High	-4,095	ref	hetero	hetero	hetero	hetero	ref
Ccrd_005870	Ccrd_v2_11622_g07	SL	GAS	7	11683491	A	G	F367S	High	-4,491	ref	hetero	ref	hetero	ref	ref
Ccrd_005870	Ccrd_v2_11622_g07	SL	GAS	7	11683483	G	A	L370F	High	-3,615	ref	ref	hetero	hetero	hetero	ref
Ccrd_021142	Ccrd_v2_14644_g10	SL	COS3*	10	17481736	C	T	R402K	High	-2,844	hetero	ref	ref	ref	ref	ref
Ccrd_021142	Ccrd_v2_14644_g10	SL	COS3*	10	17481614	G	A	P443S	High	-4,439	hetero	ref	ref	ref	ref	ref
Ccrd_021142	Ccrd_v2_14644_g10	SL	COS3*	10	17481308	G	A	L545F	High	-3,867	ref	ref	ref	hetero	ref	ref
Ccrd_021142	Ccrd_v2_14644_g10	SL	COS3*	10	17480682	C	G	G718A	High	-4,694	hetero	ref	ref	ref	ref	ref
Ccrd_021142	Ccrd_v2_14644_g10	SL	COS3*	10	17480634	T	A	D734V	High	-6,376	hetero	ref	ref	ref	ref	ref
Ccrd_008697	Ccrd_v2_20677_g14	SL	COS4	14	6939887	C	T	P389L	High	-9,656	ref	hetero	ref	ref	ref	ref
Ccrd_008697	Ccrd_v2_20677_g14	SL	COS4	14	6940097	C	A	P459H	High	-8,096	hetero	ref	ref	ref	ref	ref
Ccrd_008697	Ccrd_v2_20677_g14	SL	COS4	14	6940147	C	G	L476V	High	-2,807	ref	ref	hetero	ref	hetero	ref
Ccrd_008697	Ccrd_v2_20677_g14	SL	COS4	14	6940202	T	A	M494K	High	-5,443	ref	ref	ref	ref	hetero	ref
Ccrd_008697	Ccrd_v2_20677_g14	SL	COS4	14	6939982	G	T	1261G>T Glu421*	High	-7,546	ref	hetero	ref	hetero	ref	ref
Ccrd_008697	Ccrd_v2_20677_g14	SL	COS4	14	6939990	G	A	1269G>A Trp423*	High	-6,985	ref	hetero	ref	hetero	ref	ref
Ccrd_001147	Ccrd_v2_18204_g12	SL	COS5	12	33089827	T	C	S58P	High	-6,241	alt	alt	hetero	hetero	hetero	ref
Ccrd_001147	Ccrd_v2_18204_g12	SL	COS5	12	33091532	A	G	S424G	High	-3,144	ref	ref	hetero	hetero	ref	ref
Ccrd_001147	Ccrd_v2_18204_g12	SL	COS5	12	33091556	A	T	S432C	High	-3,637	ref	hetero	ref	ref	ref	ref
Ccrd_001147	Ccrd_v2_18204_g12	SL	COS5	12	33091560	G	A	C433Y	High	-5,211	alt	alt	hetero	hetero	hetero	ref
Ccrd_001147	Ccrd_v2_18204_g12	SL	COS5	12	33090490	CTTA	CTTAATTA	838_839 insATTA	High	-8,641	alt	hetero	ref	ref	hetero	ref
Ccrd_015146	Ccrd_v2_06405_g03	SL	PTS1	3	30879286	A	G	S175G	High	-2,695	hetero	ref	ref	ref	ref	ref
Ccrd_015146	Ccrd_v2_06405_g03	SL	PTS1	3	30879452	G	A	R230K	High	-3,716	hetero	hetero	hetero	hetero	hetero	ref

Ccrd_015146	Ccrd_v2_06405_g03	SL	PTS1	3	30881509	T	A	N335K	High	-2,675	hetero	ref	ref	ref	ref	ref
Ccrd_012076	Ccrd_v2_00705_g01	SL	PTS3	1	7809977	T	G	F614C	High	-6,15	hetero	ref	ref	ref	ref	ref
Ccrd_012076	Ccrd_v2_00705_g01	SL	PTS3	1	7810023	C	G	F629L	High	-5,007	ref	hetero	hetero	ref	hetero	ref
Ccrd_012076	Ccrd_v2_00705_g01	SL	PTS3	1	7810453	G	C	G745A	High	-2,747	ref	ref	hetero	hetero	hetero	ref
Ccrd_012076	Ccrd_v2_00705_g01	SL	PTS3	1	7797133	G	T	*805G>T Glu269*	High	-6,926	ref	ref	ref	ref	hetero	ref
Ccrd_012076	Ccrd_v2_00705_g01	SL	PTS3	1	7810911	A	G	T849A	High	-2,997	ref	hetero	hetero	ref	hetero	ref
Ccrd_012076	Ccrd_v2_00705_g01	SL	PTS3	1	7797508	TGG	TG	*1093 delG Val365fs	High	-3,589	ref	hetero	ref	ref	alt	ref
Ccrd_012076	Ccrd_v2_00705_g01	SL	PTS3	1	7815053	A	T	E1112D	High	-3	ref	alt	alt	hetero	alt	ref
Ccrd_012076	Ccrd_v2_00705_g01	SL	PTS3	1	7816801	TTC	TTCAACACGATCTC	*3804 insAACACGATCTC_fs	High	-8,428	ref	ref	ref	hetero	ref	ref
Ccrd_012074	Ccrd_v2_00703_g01	SL	PTS4	1	7766393	G	A	G435E	High	-7,442	ref	ref	hetero	ref	hetero	ref
Ccrd_012074	Ccrd_v2_00703_g01	SL	PTS4	1	7765805	G	T	*805G>T Glu269*	High	-9,355	ref	hetero	hetero	ref	hetero	ref
Ccrd_012074	Ccrd_v2_00703_g01	SL	PTS4	1	7766180	TGG	TG	*1093 delG Val365fs	High	-3,249	ref	hetero	ref	ref	ref	ref
Ccrd_012073	Ccrd_v2_00702_g01	SL	PTS5	1	7739690	C	G	A125G	High	-3,312	ref	hetero	hetero	hetero	hetero	ref
Ccrd_012073	Ccrd_v2_00702_g01	SL	PTS5	1	7740593	T	C	Y382H	High	-4,308	ref	hetero	hetero	ref	hetero	ref
Ccrd_012073	Ccrd_v2_00702_g01	SL	PTS5	1	7740765	C	G	A439G	High	-2,523	ref	hetero	hetero	hetero	hetero	ref
Ccrd_012073	Ccrd_v2_00702_g01	SL	PTS5	1	7748487	AT	A	*1652 delT Ile551fs	High	-5,338	ref	hetero	hetero	hetero	hetero	ref
Ccrd_012072	Ccrd_v2_00701_g01	SL	PTS6	1	7726104	T	A	F148L	High	-3,008	ref	ref	hetero	hetero	hetero	ref
Ccrd_012072	Ccrd_v2_00701_g01	SL	PTS6	1	7726740	G	A	M317I	High	-2,885	hetero	ref	ref	ref	ref	ref
Ccrd_012072	Ccrd_v2_00701_g01	SL	PTS6	1	7727037	T	A	D416E	High	-3,127	hetero	ref	ref	ref	ref	ref
Ccrd_012072	Ccrd_v2_00701_g01	SL	PTS6	1	7727108	C	T	A440V	High	-3,113	hetero	ref	ref	ref	ref	ref
Ccrd_012072	Ccrd_v2_00701_g01	SL	PTS6	1	7727155	G	A	D456N	High	-3,088	hetero	ref	ref	ref	ref	ref
Ccrd_012072	Ccrd_v2_00701_g01	SL	PTS6	1	7726136	TGG	TG	*478 delG Val160_fs	High	-6,286	ref	ref	hetero	hetero	hetero	ref
Ccrd_012072	Ccrd_v2_00701_g01	SL	PTS6	1	7727235	C	G	C482W	High	-3,853	ref	alt	alt	hetero	alt	ref
Ccrd_018609	Ccrd_v2_09124_g05	SL	GAO1	5	23004781	G	T	Gln462Lys	High	-7,419	alt	alt	alt	alt	alt	ref
Ccrd_018610	Ccrd_v2_09125_g05	SL	GAO2	5	23062520	A	G	L133S	High	-3,049	ref	hetero	ref	ref	hetero	ref
Ccrd_005871	Ccrd_v2_11624_g07	SL	GAO3	7	11738732	G	A	A844T	High	-3,405	ref	hetero	ref	ref	ref	ref
Ccrd_005871	Ccrd_v2_11624_g07	SL	GAO3	7	11738840	T	C	F880L	High	-3,45	hetero	hetero	hetero	hetero	hetero	hetero
Ccrd_005871	Ccrd_v2_11624_g07	SL	GAO3	7	11739140	A	T	N980Y	High	-5,797	ref	hetero	ref	ref	ref	ref

Supplementary data

Genome assembly and reconstruction data

The five genomes have been assembled at first and then reconstructed at a chromosomal scale. In particular, The A41 genome was assembled generating a total 95,970 contigs, equal to 651.6Mb. N_{50} was included in 13,964 contigs of 13.5 Kb or larger, with 35.18% GC content. Contigs were then re-ordered in 17 pseudomolecules and 7,048 scaffolds with a total length of 721.9Mb, corresponding to about 99.5% of reference genome size. The SP genome was assembled in a total 74,317 contigs, equal to 644.3Mb. N_{50} was included in 20,504 contigs of 8.9 Kb or larger, with 35.01% GC content. Contigs were then re-ordered in 17 pseudomolecules and 7,057 scaffolds with a total length of 712.3Mb, corresponding to about 98.3% of reference genome size. The VS genome was assembled in 74,740 contigs, equal to 645.9Mb. N_{50} was included in 20,425 contigs of 8.9 Kb or larger, with 35.04% GC content. Contigs were then re-ordered in 17 pseudomolecules and 7.027 scaffolds with a total length of 714.6Mb, corresponding to about 98.6% of reference genome size. The C3 genome was assembled in 77,535 contigs, equal to 652.8Mb. N_{50} was included in 19,620 contigs of 9.4 Kb or larger, with 35.28% GC content. Contigs were then re-ordered in 17 pseudomolecules and 7.715 scaffolds with a total length of 722.9Mb, corresponding to about 99.7% of reference genome size. The VT genome was assembled in 74,498 contigs, equal to 644.7Mb. N_{50} was included in 20,491 contigs of 8.9 Kb or larger, with 35.08% GC content. Contigs were then re-ordered in 17 pseudomolecules and 7.018 scaffolds with a total length of 713.1Mb, corresponding to about 98.4% of reference genome size.

Presence/Absence variants (PAVs)

The inspection of the genome-wide patterns of presence and absence variations (PAVs) revealed 346 PAV events, of which 87 were genotype-specific **(Figure S1)**. According to their ontology, enrichment analysis showed an over-representation in some GO terms **(Table S5)**. In particular, in A41, Biological Processes GO-term related to regulating host cell cycle in response to virus (GO:0060154), circumnutation (GO:0010031), multicellular organismal movement (GO:0050879) cellular response to virus (GO:0098586) were enriched. For Molecular Functions, the terms signal peptide processing (GO:0006465), ubiquitin-ubiquitin ligase activity (GO:0034450), sinapate 1-glucosyltransferase activity (GO:0050284), hydroxycinnamate 4-beta-glucosyltransferase activity (GO:0047218), glucuronosyltransferase activity (GO:0015020) were observed as enriched in A41. Considering the Cellular Component, the terms cortical microtubule, transverse to long axis (GO:0010005), cortical microtubule (GO:0055028),nuclear chromosome (GO:0000228) and external encapsulating structure (GO:0030312) showed to be enriched in A41. In plant, PAVs are less studied than animals, but their significance has been revealed by a few recent papers. Tan et al.[10] reported that PAVs could affect nearly 10% of the gene space in *Arabidopsis* and hence have been also adopted as molecular tools to scan the phenotypic variation in a number of accessions[11]. Enrichment analysis for PAV showed a slight over-representation in some GO terms in A41, specifically in relation to response to stress related GO terms (virus and light). These results were consistent with previous reports in soybean, in which the structural variants in forms of PAVs, were found to localize in gene clusters involved in defense responses[12]. However, PAVs may directly change the copies of

the defense response genes and could have a profound effect on plant stress resistance by altering the gene dosage[13]. Stress resistance genes are essential for the survival of the species and, therefore, they have also been the object of high selective pressure during domestication and breeding.

miRNA and target genes

Putative miRNA target gene enrichment analyses with AGRIGO[3] for each line, revealed significant enrichment for some GO terms **(File S3**); on the other side, no significant GO enrichments were found for VT. For each genotypes, the top ranked enrichments observed for processes were: GO:0016070 (RNA metabolic process) in 2C, GO:0009808 (lignin metabolic process) in A41, GO:0032774 (RNA biosynthetic process) in VS, GO:0080090 (regulation of primary metabolic process) in C3, and GO:0009719 (response to endogenous stimulus) for SP. With respect to functions (F), enrichments were observed in: GO:0003676 (nucleic acid binding) for 2C, GO:0005507 (copper acid binding) for A41, GO:0003677 (DNA binding) in VS, GO:0003676 (nucleic acid binding) in C3, and GO:0003677 (DNA binding) for SP. For components (C) enrichments were present for: GO:0043231 (membrane-bounded organelle) in 2C, GO:0005576 (extracellular region) in A41, GO:0005634 (nucleus) in VS, GO:0003677 (DNA binding) in VS, GO:0005634 (nucleus) in C3, and GO:0005634 (nucleus) in SP. Comparisons of GO terms enrichment (AGRIGO SEACOMPARE) applied on the five genotypes showed that just one GO term (GO:0005634: nucleus) was shared among the five genotypes (**Table S9**). On the other side, 17 biological process and 1 molecular function GO terms were shared among 2C, A41, VS and C3, including the significantly enriched GO:0003676 (nucleic acid binding), GO:0006350 (transcription), and GO:0045449 (regulation of transcription). Finally, GO:0009808 (lignin metabolic process) and GO:0046271 (phenylpropanoid catabolic process) were significantly enriched 2C, A41 and C3. The REVIGO[1] summarization of enriched terms for biological process, cellular component and molecular function, obtained by removing redundant GO terms, are reported in **Figures S2**, **S3** and **S4**, respectively.

Supplementary literature

1. Supek, F., Bošnjak, M., Škunca, N. & Šmuc, T. REVIGO summarizes and visualizes long lists of gene ontology terms. *PLoS One* **6,** e21800 (2011).
2. Li, L., Stoeckert, C. J. & Roos, D. S. OrthoMCL: identification of ortholog groups for eukaryotic genomes. *Genome Res.* **13,** 2178–89 (2003).
3. Du, Z., Zhou, X., Ling, Y., Zhang, Z. & Su, Z. agriGO: a GO analysis toolkit for the agricultural community. *Nucleic Acids Res.* **38,** W64–70 (2010).
4. de Lima Morais, D. A. *et al.* SUPERFAMILY 1.75 including a domain-centric gene ontology method. *Nucleic Acids Res.* **39,** D427–34 (2011).
5. Jones, P. *et al.* InterProScan 5: genome-scale protein function classification. *Bioinformatics* **30,** 1236–40 (2014).
6. Lamesch, P. *et al.* The Arabidopsis Information Resource (TAIR): improved gene annotation and new tools. *Nucleic Acids Res.* **40,** D1202–10 (2012).
7. Mathelier, A. & Carbone, A. MIReNA: finding microRNAs with high accuracy and no learning at genome scale and from deep sequencing data. *Bioinformatics* **26,** 2226–34 (2010).
8. Bonnet, E., He, Y., Billiau, K. & Van de Peer, Y. TAPIR, a web server for the prediction of plant microRNA targets, including target mimics. *Bioinformatics* **26,** 1566–8 (2010).
9. Scaglione, D. *et al.* The genome sequence of the outbreeding globe artichoke constructed de novo incorporating a phase-aware low-pass sequencing strategy of F1 progeny. *Sci. Rep.* **6,** 19427 (2016).
10. Tan, S. *et al.* Variation of presence/absence genes among Arabidopsis populations. *BMC Evol. Biol.* **12,** 86 (2012).
11. Salathia, N. *et al.* Indel arrays: an affordable alternative for genotyping. *Plant J.* **51,** 727–37 (2007).
12. McHale, L. K. *et al.* Structural variants in the soybean genome localize to clusters of biotic stress-response genes. *Plant Physiol.* **159,** 1295–308 (2012).
13. Cook, D. E. *et al.* Copy number variation of multiple genes at Rhg1 mediates nematode resistance in soybean. *Science* **338,** 1206–9 (2012).

Supplementary file list

- **Supplementary file 1 -** reciprocal best-hit (RBH) analysis table.
- **Supplementary file 2 –** Identified miRNAs in the 6 genotypes using MIReNA[7], together with pre-miRNA and miRNA sequences and their genomic position.
- **Supplementary file 3 -** GO term enrichment analysis for the target miRNA genes in the 6 genotypes.
- **Supplementary file 4 -** RGAs identified by Blastp against *Arabidopsis* unique RGAs proteins, together with the identified domains using HMMer.
- **Supplementary file 5 -** Phylogenetic trees for the RGAs gene classes (RLK, RLP, NB and other-KTM) in the 6 genotypes.
- **Supplementary file 6 -** Whole SNP/indel dataset (vcf file).

Genome reconstruction in *Cynara cardunculus* taxa gains access to chromosome-scale DNA variation

Alberto Acquadro[1], Lorenzo Barchi[1], Ezio Portis[1,*], Giulio Mangino[1], Danila Valentino[1], Giovanni Mauromicale[2], Sergio Lanteri[1]

Addresses

[1] DISAFA, Plant Genetics and Breeding, University of Torino, Grugliasco, Italy.
[2] Dipartimento di Agricoltura, Alimentazione e Ambiente (Di3A), University of Catania, Catania, Italy.
[*] Corresponding author.

- Supplementary file 5 - Phylogenetic trees for the RGAs gene classes (RLK, RLP, NB and other-KTM) in the 6 genotypes.

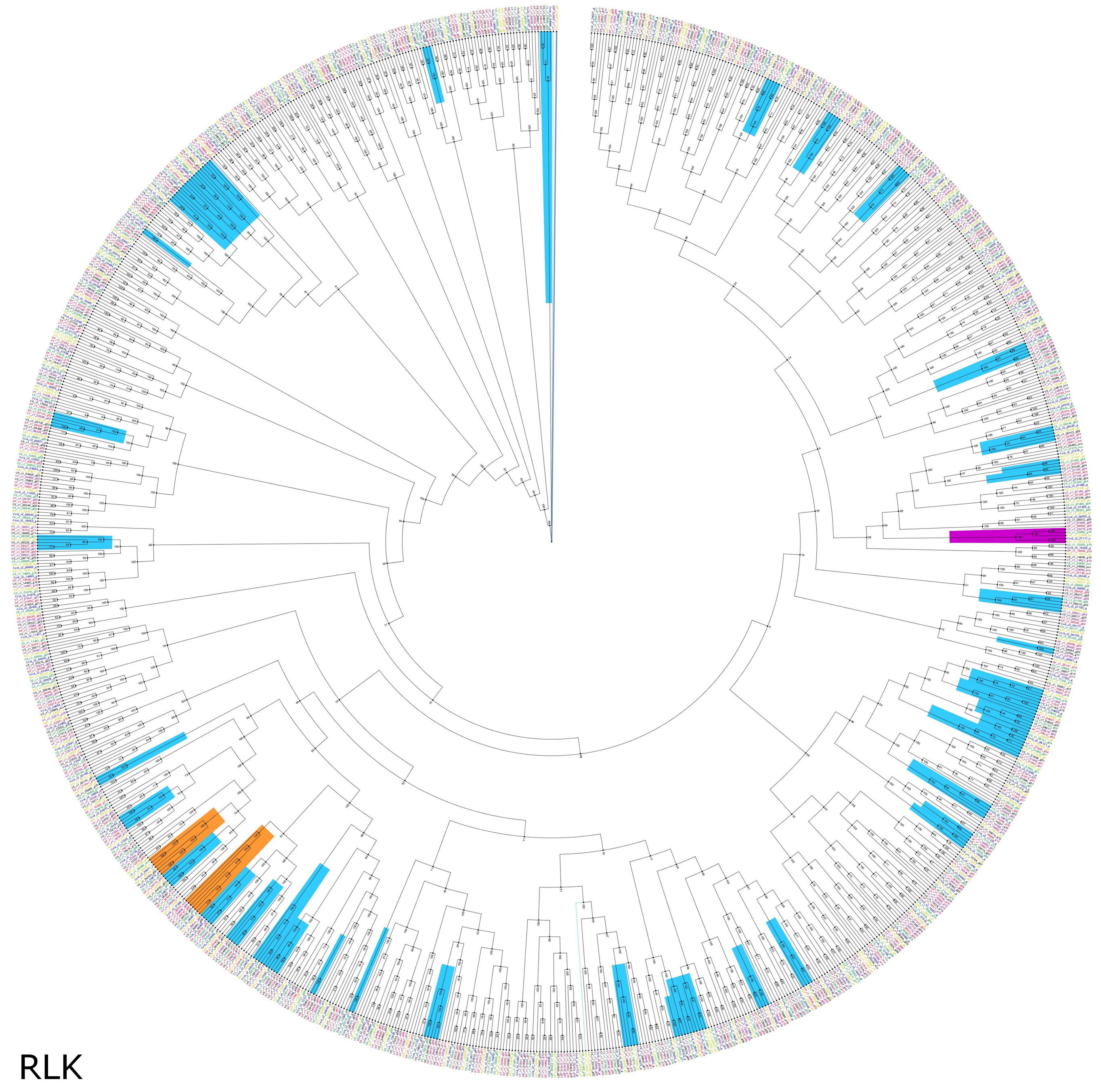

RLK

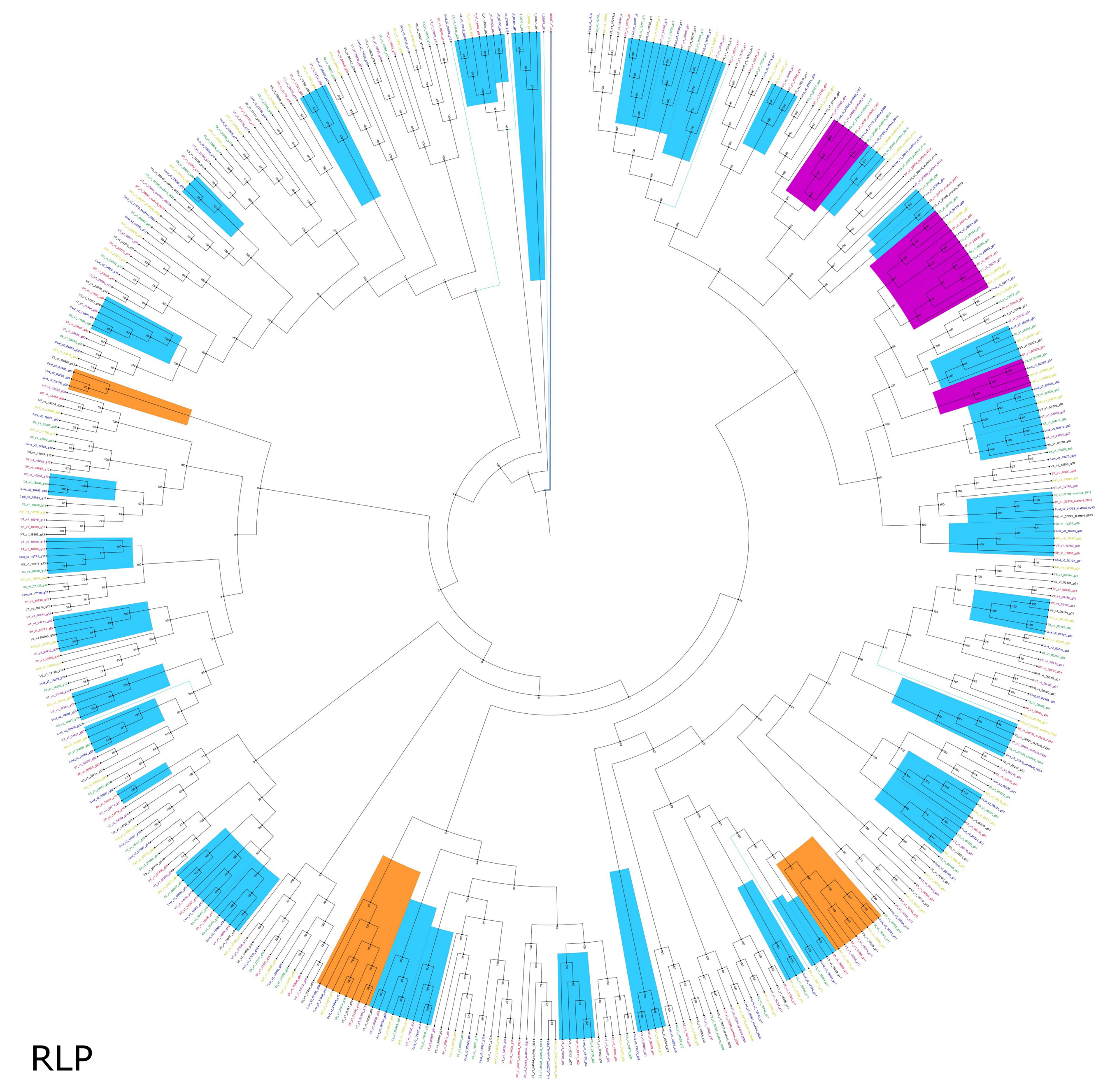

RLP

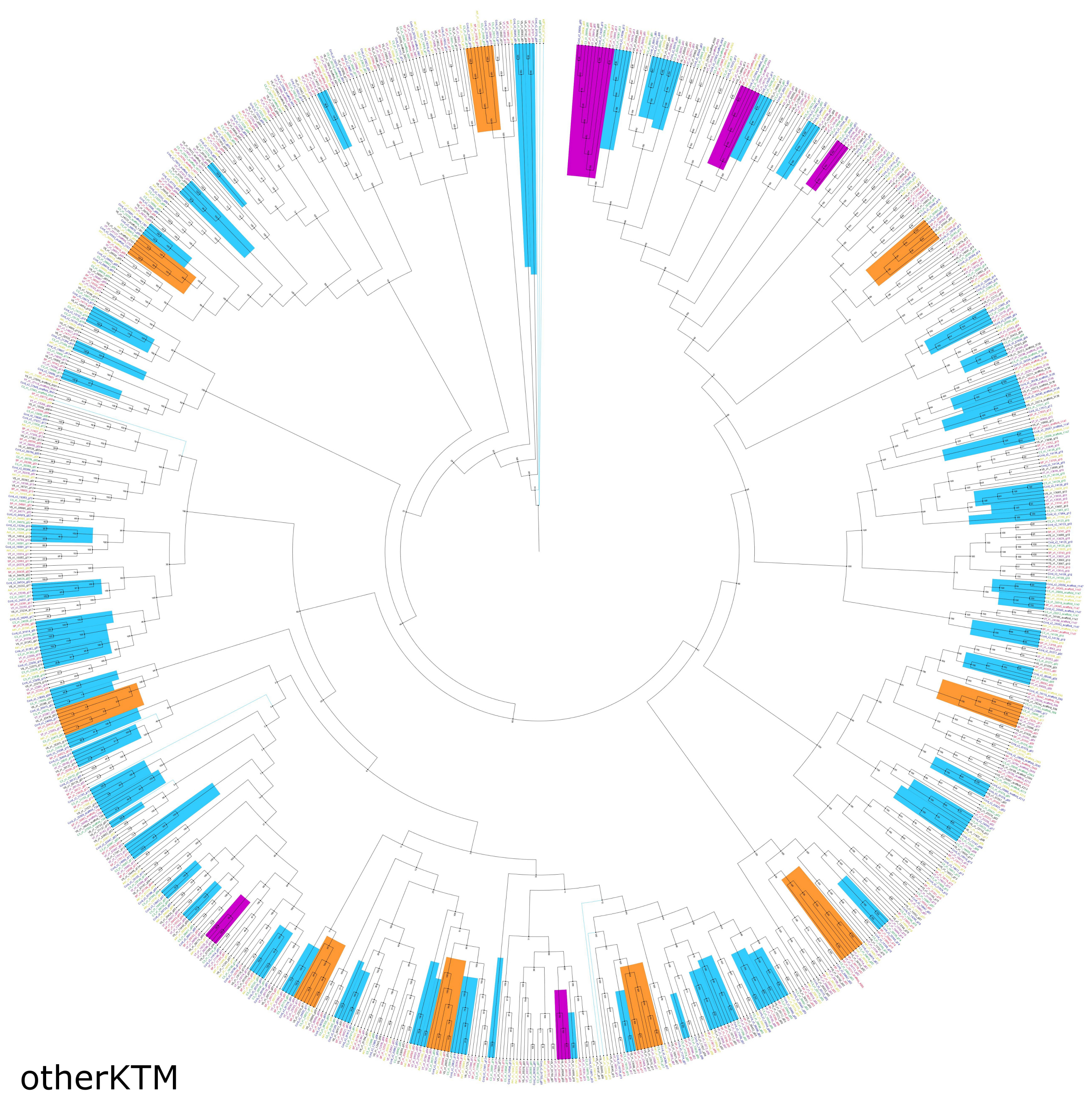

otherKTM

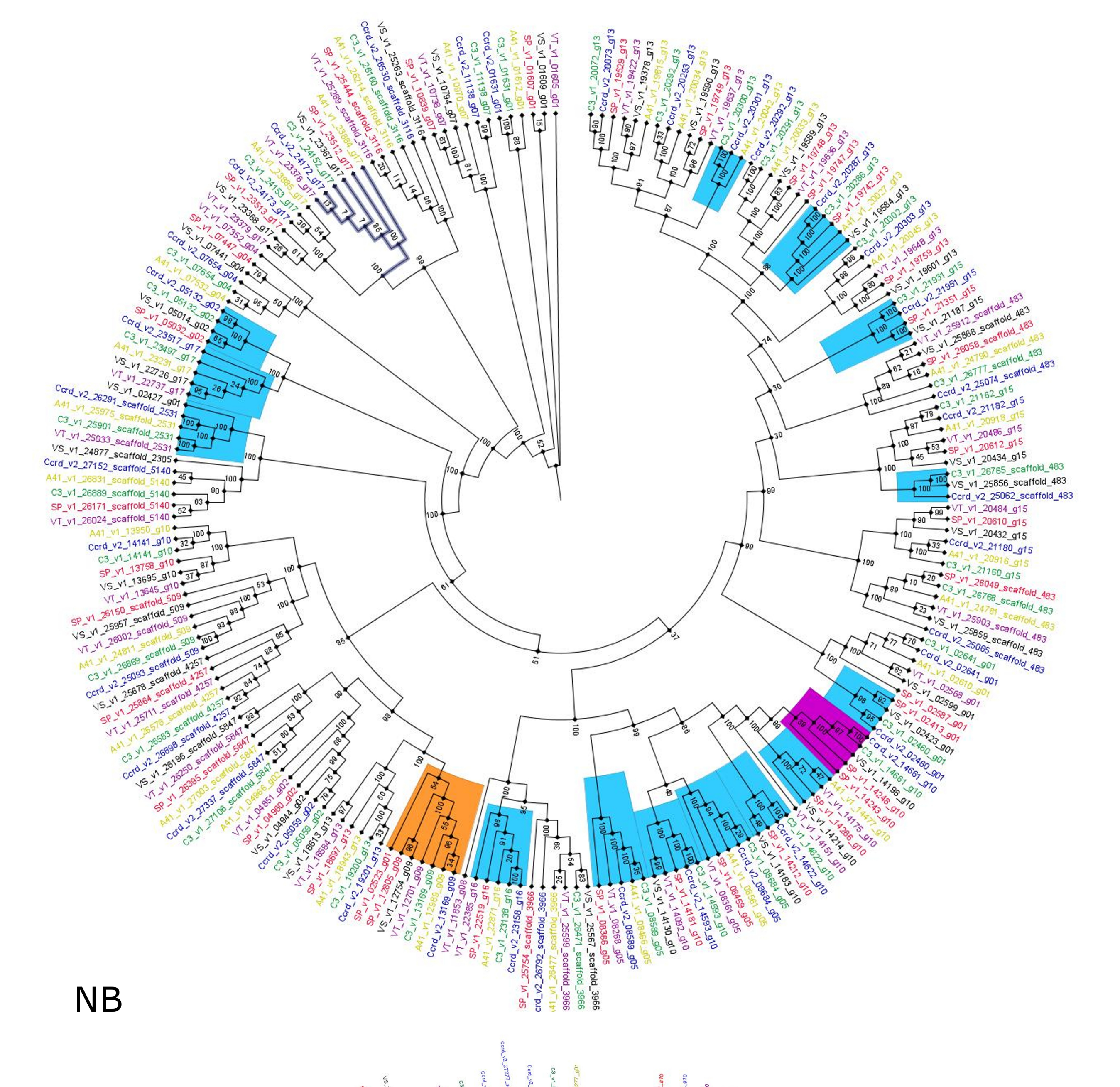

NB

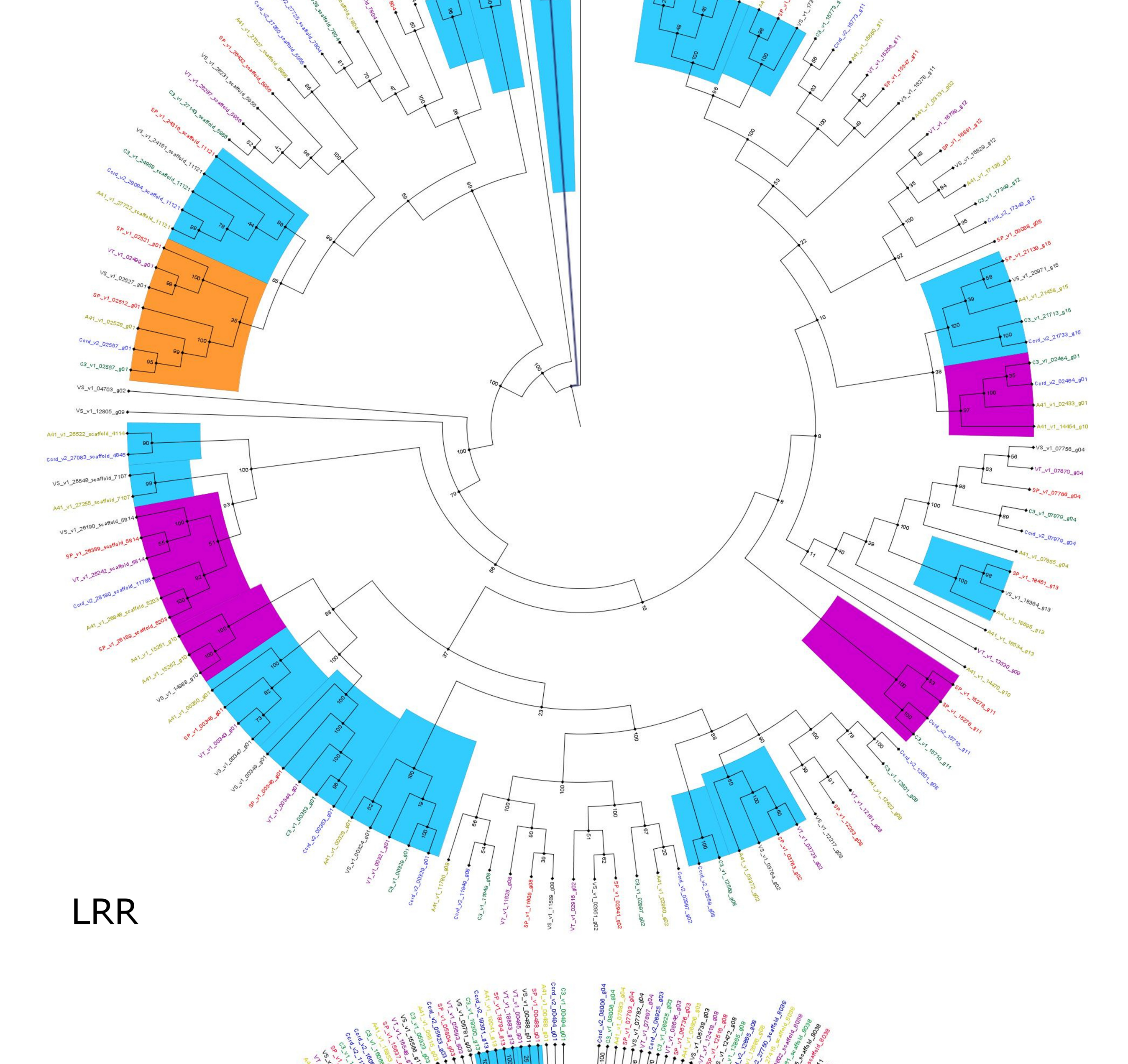

LRR

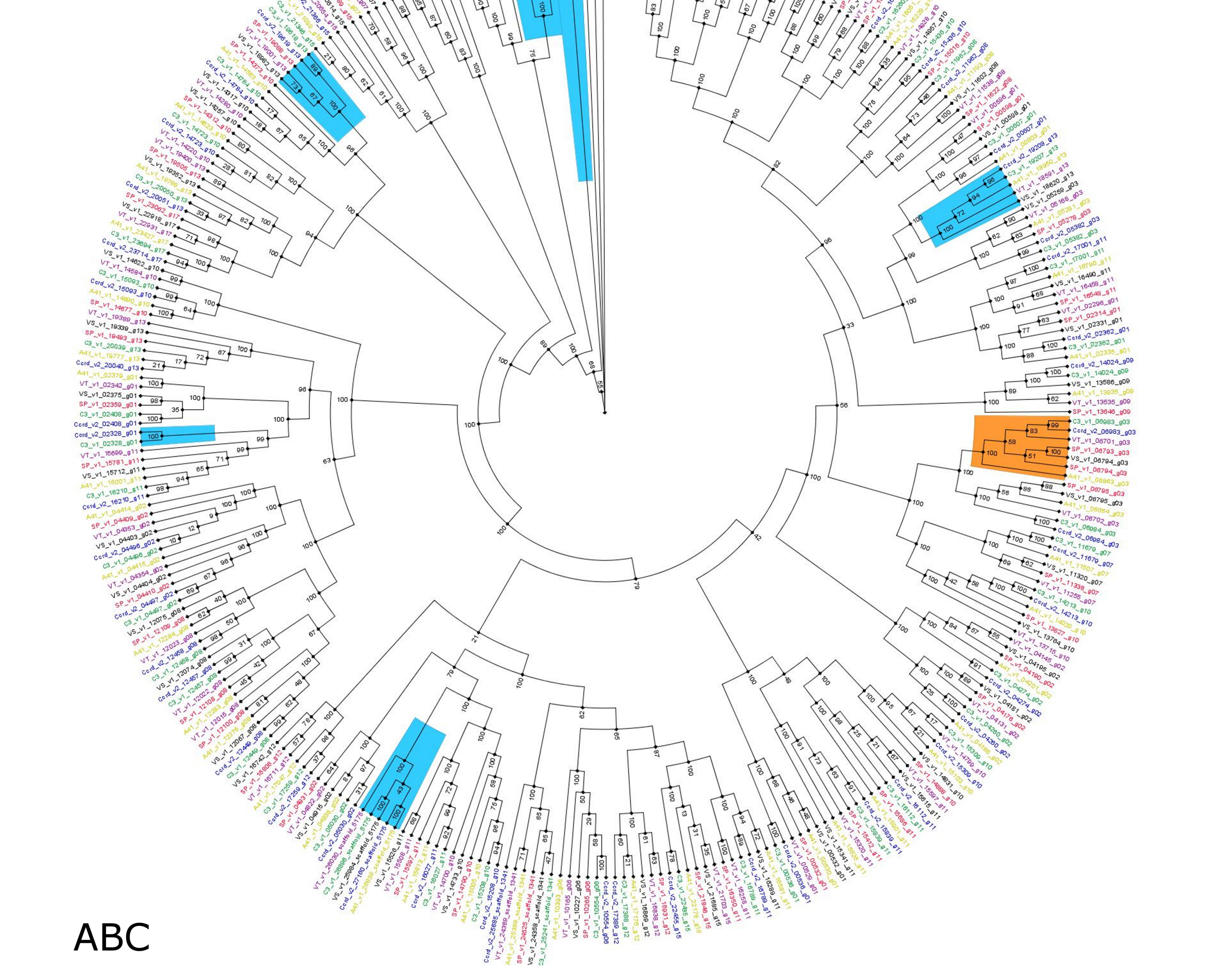

ABC
